# A Soft‐Soft Contact Triboelectric Nanogenerator with a Ternary Four‐Phase Structure for Self‐Powered High‐Efficiency Dust Removal on Mars

**DOI:** 10.1002/advs.202502956

**Published:** 2025-04-17

**Authors:** Fei Yang, Zheping Wang, Boyi Xu, Yifan Lu, Xuyan Hou, Jinsui Xu, Zhijie Xie

**Affiliations:** ^1^ National Key Laboratory of Aerospace Mechanism School of Mechatronics Engineering Harbin Institute of Technology Harbin 150001 China; ^2^ Wu Xianming School of Intelligent Engineering South China University of Technology Guangzhou 510641 China; ^3^ College of mechanical and electrical engineering Northeast Forestry University Harbin 150042 China

**Keywords:** dust removal, mars, self‐powered, ternary dielectric triboelectrification, triboelectric nanogenerator

## Abstract

Dust removal is essential for sustaining energy supplies in Mars exploration missions and base facilities. Existing devices typically depend on external power supplies, which may be inadequate in the harsh Martian environment. This paper proposes a novel wind‐driven ternary four‐phase soft‐soft contact triboelectric nanogenerator (FPS‐TENG) for dust removal. The device's double‐stacked soft‐soft contact structure outputs high‐voltage electricity in four phases, generating a traveling wave electric field on the electrode surface. This field prompts dust particles to align with the wave's direction, enabling automatic dust removal without an external power supply. The FPS‐TENG, as a ternary stacked soft‐soft contact TENG, achieves output voltages that are 354% and 185% higher than those of conventional binary and ternary TENGs, respectively. Compared with existing dust removal devices, this device can achieve a dust removal efficiency of up to 91.8% for simulated Martian dust, even at lower voltages, without requiring the repositioning of solar panels during the cleaning process. Experimental results validate the system's operation in the Martian environment. This work provides a self‐powered, high‐efficiency method for dust removal that holds significant promise for promoting wider space exploration and supporting potential future Martian settlements.

## Graphical abstract

The frequent dust storms and charged dust particles on Mars pose significant challenges to the power generation efficiency and service life of solar panels during exploration missions. This paper proposes a novel wind‐driven ternary four‐phase soft‐soft contact triboelectric nanogenerator (FPS‐TENG) for dust removal. The device's double‐stacked soft‐soft contact structure outputs high‐voltage electricity in four phases, generating a traveling wave electric field on the electrode surface. This field prompts dust particles to align with the wave's direction, enabling automatic dust removal without an external power supply.

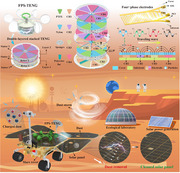

## 1 Introduction

Space exploration acts as a major driver of technological advancements. It has not only greatly expanded the understanding of the vast universe but also unveiled opportunities for utilizing essential space resources.^[^ ^1^, ^2^ ^]^ Mars, which closely mirrors Earth's environmental conditions, remains at the forefront of international deep‐space missions, intriguing scientists with its potential for extraterrestrial life and habitable environments.^[^ ^3^ ^]^ The distinct environmental challenges on Mars, especially frequent dust storms and charged particles, significantly reduce the efficiency and longevity of solar panels, thereby jeopardizing the sustainability of missions.^[^ ^4^, ^5^ ^]^ Consequently, innovating effective dust mitigation technologies is crucial for maintaining reliable operations and energy provision on Mars.

Addressing the dust accumulation on solar panels, several researchers have explored and deployed diverse cleaning strategies. These strategies include not only passive dust removal techniques but also active dust removal techniques. Passive dust removal techniques primarily rely on material properties to reduce dust accumulation, such as dust‐resistant coatings.^[^ ^6^, ^7^ ^]^ These coatings are often hydrophilic or antistatic, making it difficult for dust to adhere to the surface of the panels or allowing it to be easily removed by rainwater. Active dust removal techniques mainly include mechanical brushing,^[^ ^8^, ^9^ ^]^ high‐pressure water jet cleaning,^[^ ^10^ ^]^ ultrasonic cleaning,^[^ ^11^ ^]^ and electrodynamic screen (EDS) removal.^[^ ^12^, ^13^, ^14^ ^]^ Mechanical brushing effectively removes dust but may lead to gradual surface degradation. While high‐pressure water jet cleaning is widely used on Earth, its application on Mars is limited due to the scarcity of water resources. Ultrasonic cleaning, which uses sound vibrations to dislodge dust, demands extra energy—a significant challenge in Mars' restricted energy environment. Similarly, EDS technology uses alternating electric fields to clear dust from solar panels and spacesuits but is constrained by the available power supply. These conventional methods, dependent on sophisticated machinery and extra power, escalate the complexity and operational costs of missions. Thus, the development of a self‐sustaining dust removal technology is essential for the sustained functionality of Martian exploratory equipment.

The triboelectric nanogenerator (TENG) marks a significant breakthrough in self‐powered dust removal technology. This device transforms mechanical energy into electrical energy via contact electrification and electrostatic induction, capturing energy from a variety of sources, such as human motion,^[^ ^15^, ^16^ ^]^ wind energy,^[^ ^17^, ^18^, ^19^ ^]^ and ocean wave energy.^[^ ^20^, ^21^ ^]^ Ma et al.^[^ ^17^ ^]^ developed a noncontact CBO‐TENG that boasts a dust removal efficiency of up to 79.2%, achieved by generating sustained vibrations through instantaneous impacts. Heo et al.^[^ ^19^ ^]^ found that an RTENG, when exposed to wind speeds of 28 m s^−1^, recovered over 90% of the original solar energy efficiency, substantially boosting solar panel power output. Ding et al.^[^ ^18^ ^]^ engineered a self‐powered automatic dust removal system, composed of a wind‐driven electret generator, a voltage multiplication circuit, and a dust removal unit, which reached a dust removal efficiency exceeding 90% at wind speeds as low as 1.6 m s^−1^. Although these triboelectric dust removal devices demonstrate their dust removal capability, their mechanism is based on standing wave dust removal. In a standing wave electric field, charged particles move back and forth under the influence of the field without directional movement, making it ineffective at removing fine dust from the surface of solar panels. As a result, their performance is often limited by the tilt angle of the solar panels. Consequently, developing a self‐powered dust removal device that operates effectively in the Martian environment without being affected by the solar panels’ tilt angle is critical.

This study proposes a novel self‐powered dust removal technology utilizing a wind‐driven ternary four‐phase soft‐soft contact triboelectric nanogenerator (FPS‐TENG) designed for cleaning solar panels during Mars missions. The FPS‐TENG employs wind‐driven rotary motion to generate a quadraphasic alternating voltage, generating a traveling wave electric field across the electrodes that efficiently removes charged dust particles. Its distinctive ternary four‐phase soft‐soft contact rotary structure not only significantly enhances output voltage density but also ensures robust dust removal. The output voltage of the FPS‐TENG (stacked) is 354% and 185% higher than that of the CR‐TENG and PFR‐TENG, respectively. The FPS‐TENG is driven by natural wind on Mars and generates sufficient power to remove dust from solar panels without requiring an external power source. Even at relatively low voltages, it achieves a dust removal efficiency of up to 91.8% for simulated Martian dust. This self‐powered, high‐efficiency dust removal technology has significantly lower energy consumption and maintenance requirements compared to existing dust removal technologies that rely on external power sources. It also outperforms self‐powered standing wave dust removal devices (dust removal efficiency below 80%) and is unaffected by the tilt angle of solar panels, enhancing the energy self‐sufficiency and environmental adaptability of Martian exploration missions. Furthermore, this study investigates the dust removal effects of the FPS‐TENG for different wind speeds and panel tilt angles, experimentally verifying its effectiveness in a simulated Martian environment. This development signifies a pivotal step forward in dust removal technology, poised to significantly impact future Mars and space exploration missions.

## 2 Results and Discussion

### 2.1 The Design and Principle of FPS‐TENG

**Figure 1:**
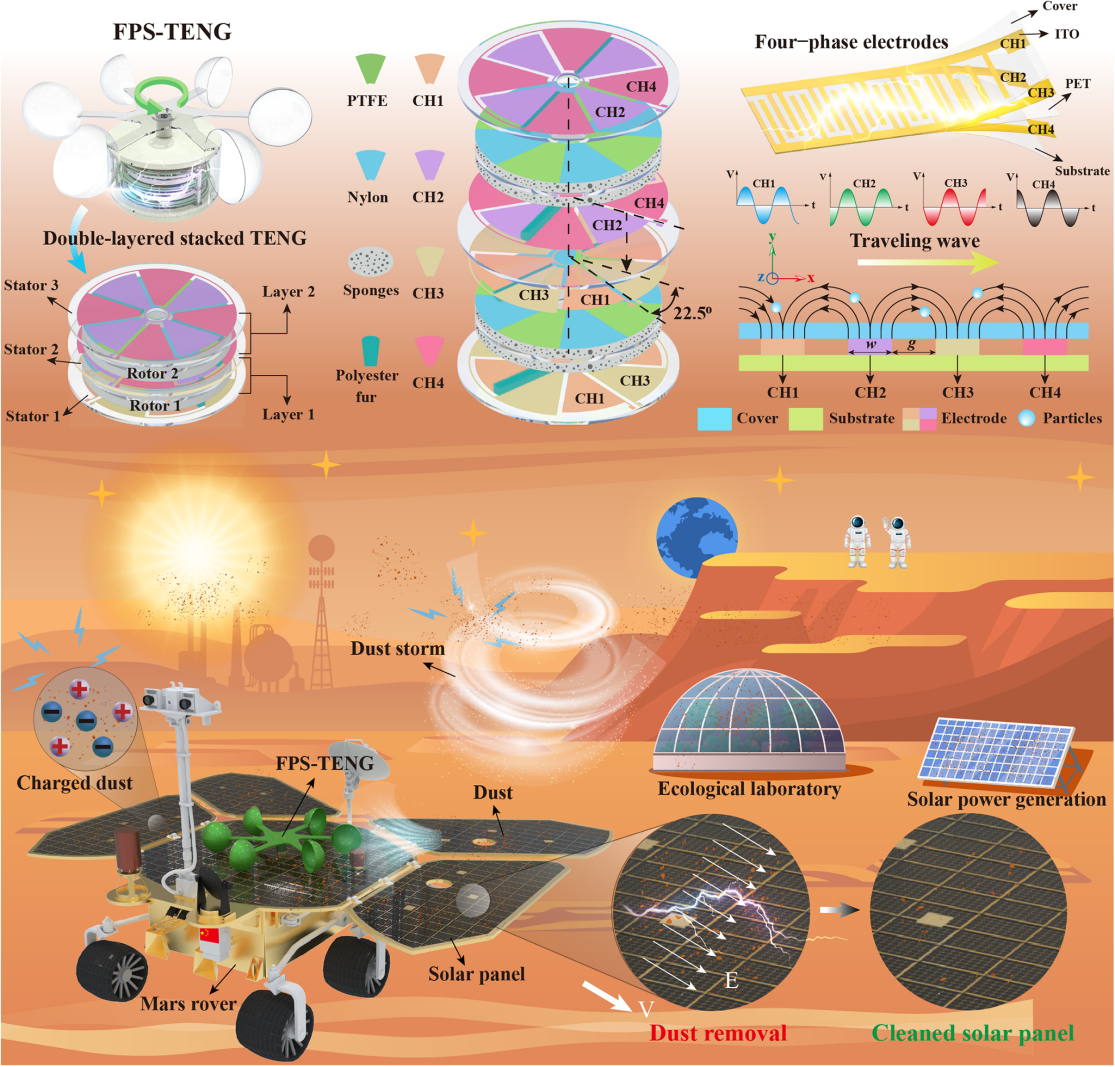
Schematic and application of FPS‐TENG dust removal. A) Schematic diagram of the FPS‐TENG dust removal device. B) Double‐layer stacked structure of the friction layer in FPS‐TENG. C) Exploded view of FPS‐TENG. D) Schematic diagram of the four‐phase electrode structure. E) Principle of dust removal by traveling wave electric field. F) FPS‐TENG utilizes wind energy to remove dust from Mars rovers and Martian facilities.

Figure 1A displays the design of a ternary four‐phase soft‐soft contact rotary triboelectric nanogenerator, FPS‐TENG, which is divided into two main sections: the upper section contains the wind cup, and the lower section houses the four‐phase friction layer. Wind rotation activates the wind cup, which in turn triggers the rotation of the friction layer, generating a four‐phase voltage output. Figure 1B presents a diagram of the double‐layer stacked structure of FPS‐TENG, which includes three stators and two rotors. This double‐layer configuration aims not only to expand the contact area, enhancing the output voltage, but also to offset the upper stack structure by 22.5° relative to the lower stack, enabling the generation of a four‐phase voltage. The specific reason for selecting a 22.5° phase offset angle is based on our design objective of achieving four‐phase voltage output, with a phase angle difference of 90° between each phase. Taking CH1 and CH3 as examples, both consist of four sectorial structures, with a phase angle difference of 180° between CH1 and CH3. The structural installation offset angle is calculated as 360°/8 = 45°. To ensure a phase angle difference of 90° between CH3 and CH2, the installation offset angle is set to 45°/2 = 22.5°. Thus, we selected a 22.5° offset angle to ensure that the upper stacking layer and lower stacking layer in the double‐stacked structure differ by one‐quarter of a cycle, enabling the output of four‐phase voltage.

The exploded view of this intricate stacked structure is shown in Figure 1C. Sector‐shaped electrodes installed on acrylic boards form the stators; Stator 1 is equipped with electrodes CH1 and CH3, Stator 2 positions electrodes CH1 and CH3 on the bottom and electrodes CH2 and CH4 on the top, while Stator 3 accommodates electrodes CH2 and CH4. These configurations ensure that the angular separation between CH1 and CH3, as well as CH2 and CH4, is 45°, which results in a phase angle difference of 180° for the voltage outputs of CH1 and CH3 compared to CH2 and CH4, and a 90° phase difference between CH1 and CH2.

Polyester fiber brushes, situated in the notches between two sets of sector‐shaped electrodes on the stator, facilitate the contact necessary for energy generation. The rotor, connected to the rotating shaft via key slots, consists of an acrylic plate, sponge, PTFE film, and nylon film. The placement of PTFE and nylon films on alternate sides of the acrylic plate enables the brush to alternate between positive and negative charges upon contact, establishing a ternary material system. Therefore, the interactions between the stator and the rotor through the contact of the brush with PTFE and nylon on the sponge surface represent a soft‐soft contact mechanism. The construction processes for the FPS‐TENG's stator and rotor are shown in Figures S1 and S2 (Supporting Information), showing their physical dimensions.

A four‐phase electrode, depicted in Figure 1D and connected to the solar panel, facilitates the FPS‐TENG's four‐phase voltage output. This electrode is constructed from five layers: a transparent cover film (PET), ITO electrodes CH1 and CH3, an insulating isolation layer (PET), ITO electrodes CH2 and CH4, and a substrate (PET). The electrode composite structure is made from transparent materials, ensuring good light transmittance. The fabrication process of the four‐phase electrode is shown in Figure S3 (Supporting Information). Figure 1E illustrates the dust removal mechanism via a four‐phase traveling‐wave electric field generated by the electrode's voltage output. The voltages are phase‐shifted by 90°, forming sine waveforms.

The input voltages at positions *x* = 0, *x* = *w*+*g*, *x* = 2(*w*+*g*) and *x* = 3(*w*+*g*) on the electrode are labeled *V*_CH1_, *V*_CH2_, *V*_CH3_ and *V*_CH4_ respectively. These voltages are calculated using Equation (1).

(1) $$\left\{\begin{array}{c} V_{\mathrm{CH}1}=V_{0}\cos\left(\omega t\right)\\ V_{\mathrm{CH}2}=V_{0}\cos\left(\omega t-\frac{\pi }{2}\right)\\ V_{\mathrm{CH}3}=V_{0}\cos\left(\omega t-\pi \right)\\ V_{\mathrm{CH}4}=V_{0}\cos\left(\omega t-\frac{3\pi }{2}\right) \end{array}\right.$$

Here, *w* represents the width of the electrode, *g* is the spacing between electrodes, *V*_0_ is the peak alternating voltage, and *ω* is the angular frequency of the alternating voltage.

The electrode's electric potential wave *V*(*x,y,t*) is modeled as the superposition of two harmonics, as shown in Equations (2) and (3).^[^ ^22^ ^]^

(2) $$V_{4}\left(x,y,t\right)\approx W_{4\mathrm{for}}+W_{4\mathrm{back}}$$

(3) $$\begin{array}{ccc} V_{4}\left(x,y,t\right) & \approx & V_{0}\left[A_{41}e^{-\frac{2\pi }{\lambda_{4}}y}\cos\left(\frac{2\pi }{\lambda_{4}}x-\omega t\right)\right.\\ & & \left.+A_{43}e^{-\frac{6\pi }{\lambda_{4}}y}\cos\left(\frac{6\pi }{\lambda_{4}}x+\omega t\right)\right] \end{array}$$

In the above equations, λ = 4(*w*+*g*) represents the geometric periodic wavelength. *W*_for_​ (the forward wave, moving in the positive direction of the *x*‐axis) is the first harmonic, while *W*_back_ (the reverse wave, traveling in the negative direction of the *x*‐axis) serves as the third harmonic, with both harmonics traveling in opposite directions. *a*_1_ and *a*_3_ are the Fourier coefficients for these harmonics, respectively.

Charged dust particles, influenced by Coulomb and dielectrophoretic forces within the traveling wave electric field, move along the electrodes as the field progresses. This process is analogous to how tidal waves propel debris along a shore, efficiently transporting dust away from the cleaning area and thus clearing it from the surface of the solar panel.

Figure 1F illustrates how the FPS‐TENG utilizes wind energy to remove dust from Mars rovers and Martian facilities. Dependence on solar panels for electrical power is crucial for Mars exploration and the establishment of Martian bases. Martian dust, charged by the solar wind, cosmic rays, and other environmental factors, clings to the solar panels due to electrostatic forces. Additionally, dust storms on Mars can rapidly deposit a significant layer of dust on the panels and glass surfaces, intensifying this problem. The FPS‐TENG efficiently removes this dust by transforming wind energy into electrical energy and generating a traveling wave electric field on the panel surfaces.

This method of dust removal could be particularly effective after prolonged dust storms, where such harsh conditions might compromise the operational capabilities of Mars rovers and base facilities due to energy consumption. Traditional dust removal methods that rely on external power sources might become ineffective in such situations. Importantly, the FPS‐TENG presented in this study operates independently of an external power source, and its dust removal efficiency improves with increasing wind strength. This feature makes it a self‐reliant, effective, and sustainable option for managing dust in Martian explorations.

**Figure 2:**
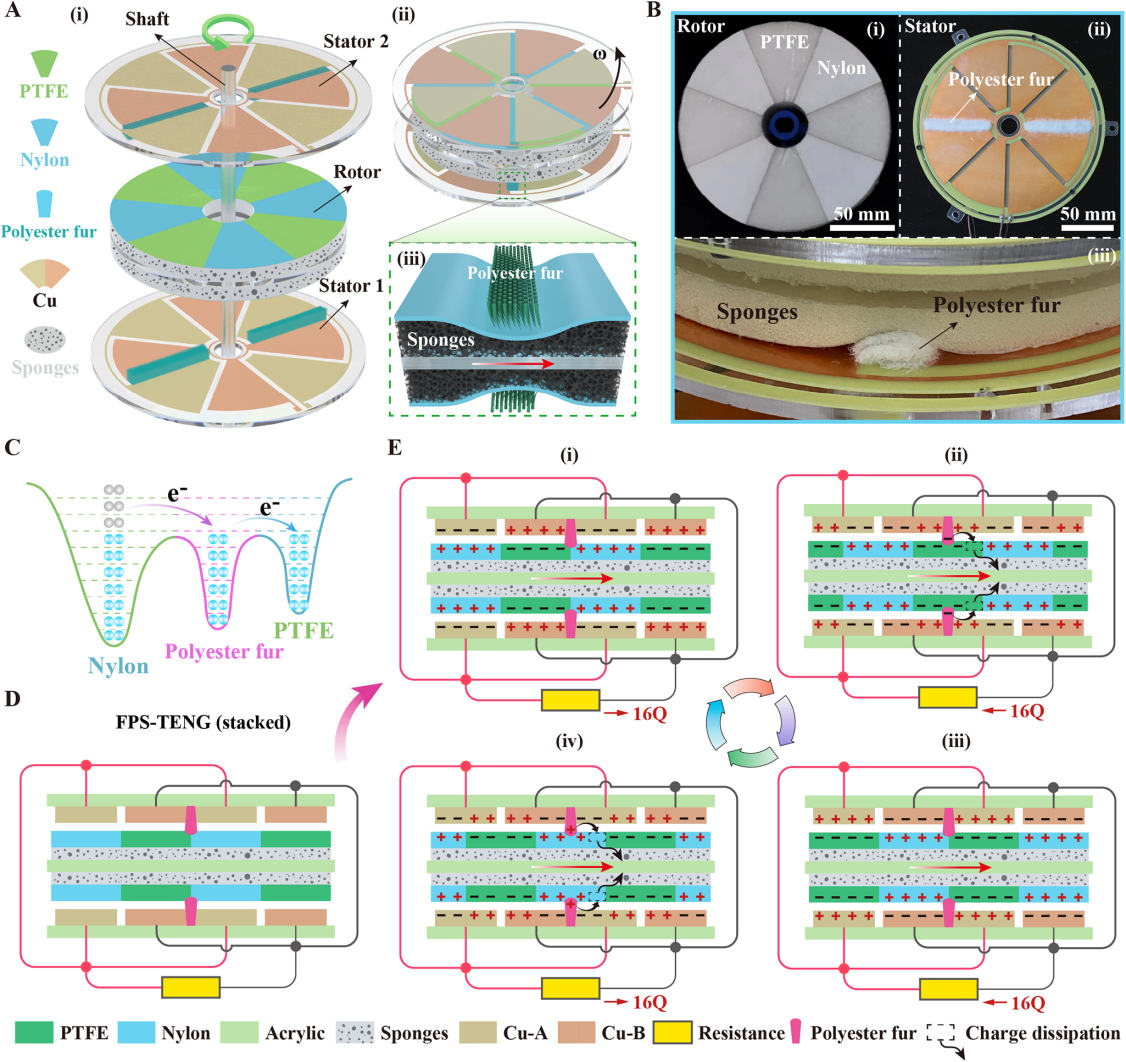
Stacked structure and working principle of FPS‐TENG. A) Exploded view of the FPS‐TENG stacked structure. B) Photograph of the rotor and stator components. C) Electron cloud potential well model for the surface charge transfer of the three types of contact triboelectric materials. D) Schematic cross‐sectional view of a single‐layer stack in FPS‐TENG. E) Working principle of the single‐layer stacked ternary FPS‐TENG.

To better illustrate the contact structure of the stator and rotor of the FPS‐TENG, this discussion utilizes the single‐layer stacked FPS‐TENG structure as an example, as shown in **Figure** **2**A. The stacked FPS‐TENG includes one rotor situated between two stators, all connected to the rotating shaft. The rotor is constructed from an acrylic plate with sponge layers attached to either side. The stator, which uses an acrylic plate as its base, features two sets of sector‐shaped copper electrodes for sensing and two brushes (polyester fur) symmetrically positioned to facilitate charge generation and transfer, as depicted in Figure 2Ai. To prevent operational air disturbances by the stator electrodes, the gap between the edges of two adjacent electrodes is maintained at 4 mm. The manufacturing process of the FPS‐TENG is described in detail in the experimental section. Figure 2Aii‐iii shows the assembly and soft contact effects of the FPS‐TENG (stacked), showing that when the brush contacts the PTFE and nylon films on the sponge, the sponge deforms, optimizing contact and minimizing the gap between the rotor and stator, which in turn enhances the output voltage. Figure 2Bi‐ii presents the physical diagrams of the rotor and stator, and Figure 2Biii depicts how the brush's contact with the sponge rotor causes the sponge to compress.

When two dissimilar materials make contact, their different electron affinities prompt electron transfer, charging the surfaces positively and negatively, respectively. The typical binary TENG structure is modified by introducing a third material, creating a ternary structure that usually consists of two triboelectric layers (PTFE, nylon) and an intermediary layer (polyester fur). The electron clouds of each layer form potential wells, with the charge affinity of the intermediary layer positioned optimally between the others to enhance charge transfer and retention (Figure 2C). The cross‐sectional view of the single‐layer FPS‐TENG (stacked) is shown in Figure 2D, where electrodes from the upper and lower layers are interconnected with wires to increase the contact area and boost the output voltage. The brush not only acts as a mediator for electron transfer but also serves as a dynamic charge pump. When the charge on PTFE and nylon begins to dissipate, the brush quickly replenishes it, thereby maintaining the generator's stable output.

Figure S4 (Supporting Information) illustrates the working principle of the FPS‐TENG before the dielectric layer reaches charge saturation. At a rotational speed of 100 rpm, the friction layer materials reach a stable state after 30 s. During this process, the polyester brush generates triboelectric charges through friction with the PTFE and nylon films. The PTFE film continuously gains electrons, while the Nylon film continuously loses electrons, causing the output voltage of the FPS‐TENG (stacked) to increase until saturation is reached (Figure S5, Supporting Information). Once the charges on the PTFE and nylon films reach saturation, the FPS‐TENG (stacked) operates as illustrated in Figure 2E.

Figure 2E shows two pairs of copper electrodes along with PTFE and nylon films. The copper electrode connected to the red wire is defined as electrode A, while the one connected to the black wire is electrode B. Due to electrostatic induction, the copper electrode A corresponding to the Nylon film carries an equal amount of negative charge, while the copper electrode B corresponding to the PTFE film carries an equal amount of positive charge. At this point, the number of positive and negative charges on the surfaces of the two electrodes is equal, as shown in Figure 2Ei.

As charge dissipation occurs, the charges on the PTFE film dissipate into the air. However, with the rotation of the rotor, friction between the PTFE film and the brush replenishes the dissipated charges in a timely manner. Simultaneously, due to electrostatic induction, electrons continuously flow from copper electrode A to copper electrode B, as shown in Figure 2Eii.

As the rotor continues to rotate, when the PTFE film and Nylon film overlap with copper electrodes A and B, respectively, the electron flow from copper electrode A to copper electrode B stops. At this point, the number of positive and negative charges on the two electrodes becomes equal again, but with opposite polarity compared to the previous state, as shown in Figure 2Eiii.

Similarly, as the rotor continues to rotate, the dissipated charges on the Nylon film are replenished through friction with the brush. Meanwhile, electrons continuously flow from copper electrode B to copper electrode A, as shown in Figure 2Eiv.

With continuous rotor movement, the induced charges on the upper and lower stator electrodes alternate, generating alternating voltage between the two sets of electrodes. During this process, the brush acts as a charge pump, continuously replenishing the dissipated charges. Additionally, we analyzed the charge transfer principle of the FPS‐TENG under stable operating conditions (Figure S6, Note S1, Supporting Information).

### 2.2 The Output Characteristics of FPS‐TENG

**Figure 3:**
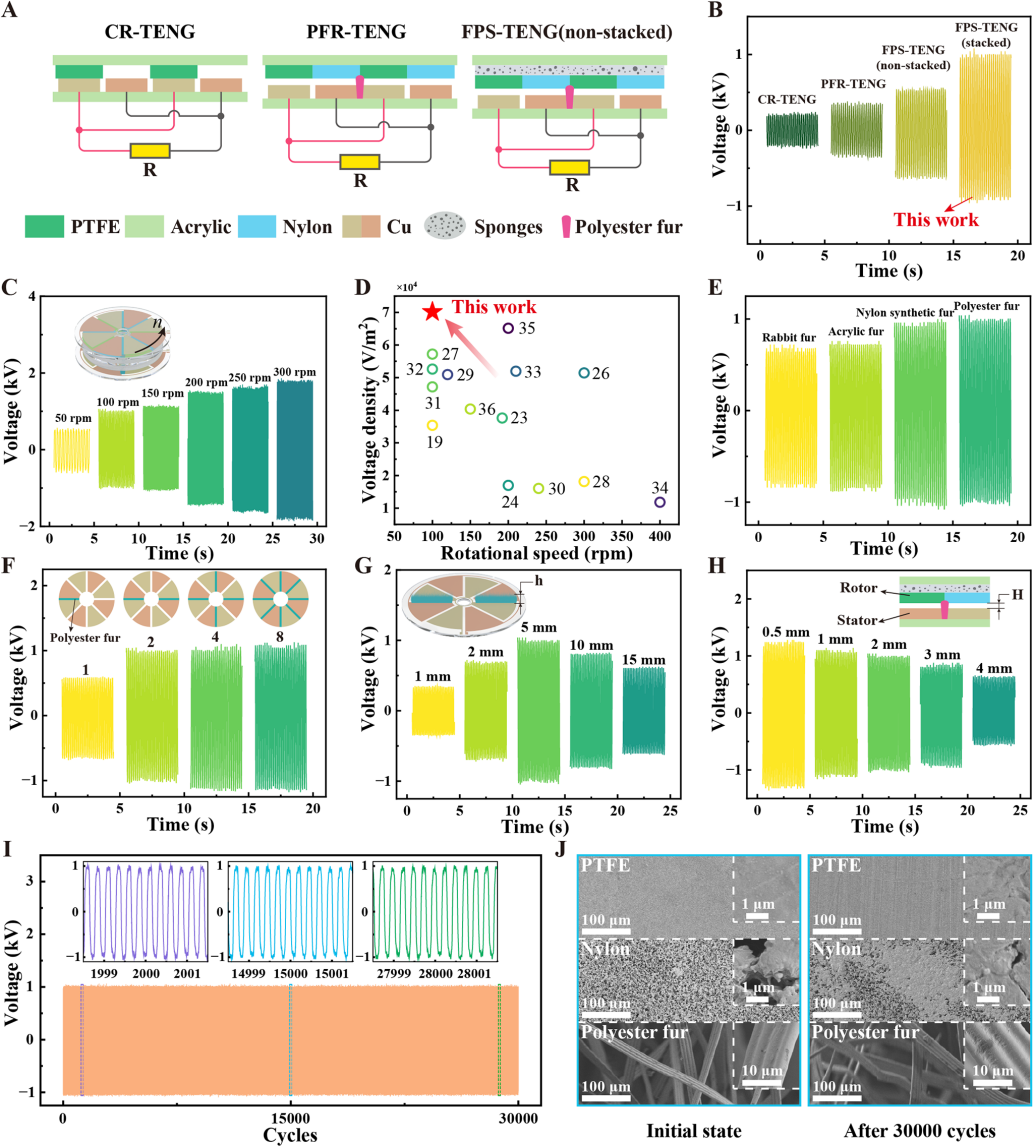
The output characteristics of FPS‐TENG. A) Schematic diagrams of three types of TENG structures. B) Comparative output performance of the four TENGs at 100 rpm. C) The output voltage of FPS‐TENG at various rotational speeds. D) Comparison of voltage density output by TENGs at different rotational speeds. E) The output voltage of FPS‐TENG with brushes made of different materials (*n* = 100 rpm). F) Influence of the number of brush groups on output voltage. G) Effect of brush height on the output voltage. H) Impact of the gap on output voltage. I) Durability test of FPS‐TENG. J) SEM images of the friction materials before and after the durability test.

To highlight the advantages of the FPS‐TENG proposed in this study, a comparative analysis was conducted between the classic binary contact rotary triboelectric nanogenerator (CR‐TENG),^[^ ^23^, ^24^ ^]^ the ternary polyester fur‐reinforced rotary triboelectric nanogenerator (PFR‐TENG),^[^ ^19^, ^25^ ^]^ as well as the non‐stacked and stacked versions of the FPS‐TENG, presented in this paper. Schematic cross‐sectional views of these four TENG models are depicted in **Figure** **3**A and Figure 2D. The CR‐TENG is characterized by a binary, hard‐hard contact without a gap between the rotor and stator, whereas the PFR‐TENG incorporates brushes as an intermediary medium in a hard‐soft contact setup. Both the non‐stacked and the stacked FPS‐TENG, the latter of which is discussed in this paper, utilize a soft‐soft contact stacked structure. Output voltage measurements under identical conditions (*n* = 100 rpm) are shown in Figure 3B, revealing that the stacked FPS‐TENG developed in this study produces the highest output voltage, surpassing the CR‐TENG and PFR‐TENG by 354% and 185%, respectively. Notably, the voltage output of the stacked FPS‐TENG is approximately twice that of the non‐stacked version (Movie S1, Supporting Information). Additionally, we designed two types of ternary stacked TENGs without polyester fur for comparison with the FPS‐TENG (stacked), as shown in Figure S7 (Supporting Information). The results indicate that the output voltage of the FPS‐TENG (stacked) with polyester fur is consistently higher than that of the structures without polyester fur. Indicating its superior voltage output capability. Figure 3C and Figure S8A,B (Supporting Information) assess how rotational speed affects the open‐circuit voltage *V*_OC_, short‐circuit current *I*_SC_, and transferred charge *Q*_SC_ of the stacked FPS‐TENG. As rotational speeds increase, *V*_OC_ and *I*_SC_ rise, though the rate of increase slows, while *Q*_SC_ diminishes. As shown in Figure 3D, a comparison of the output voltage density of previously reported rotary TENGs^[^ ^19^, ^23^, ^24^, ^26^, ^27^, ^28^, ^29^, ^30^, ^31^, ^32^, ^33^, ^34^, ^35^, ^36^ ^]^ at various rotational speeds indicates that the FPS‐TENG in this study offers a higher output voltage per unit area at lower speeds.

Figure 3E examines the effect of different brush materials on the output voltage, establishing that polyester fur yields the highest voltage output. Consequently, polyester fur was selected for the brushes in this study, with images of various fur types provided in Figure S8C (Supporting Information). Additionally, the output performance of the FPS‐TENG is influenced by the number of brushes, the height of the brushes, and the gap between the rotor and stator, as shown in Figure 3F–H. The output voltage increases with the addition of more than two brushes and reaches its peak with a brush height of 5 mm. Reducing the gap between the rotor and stator enhances the output voltage while also increasing friction, hence, a 2 mm gap is maintained for optimal performance under low damping conditions.

Figure 3I assesses the durability of the FPS‐TENG at 100 rpm, showing that the voltage remains stable after 30 000 cycles, evidencing the device's robust endurance and stability. The SEM images in Figure 3J, taken before and after the durability test, show slight bending and minor wear on the polyester fur and only minor abrasions on the PTFE and nylon films, confirming the structural and material durability of the FPS‐TENG. Figure S8D (Supporting Information) illustrates the output power of the FPS‐TENG under various external loads at 100 rpm, with the device capable of delivering a maximum output power of 8.40 mW.

### 2.3 The Output Characteristics of the FPS‐TENG in Earth's and Mars' Environments

**Figure 4:**
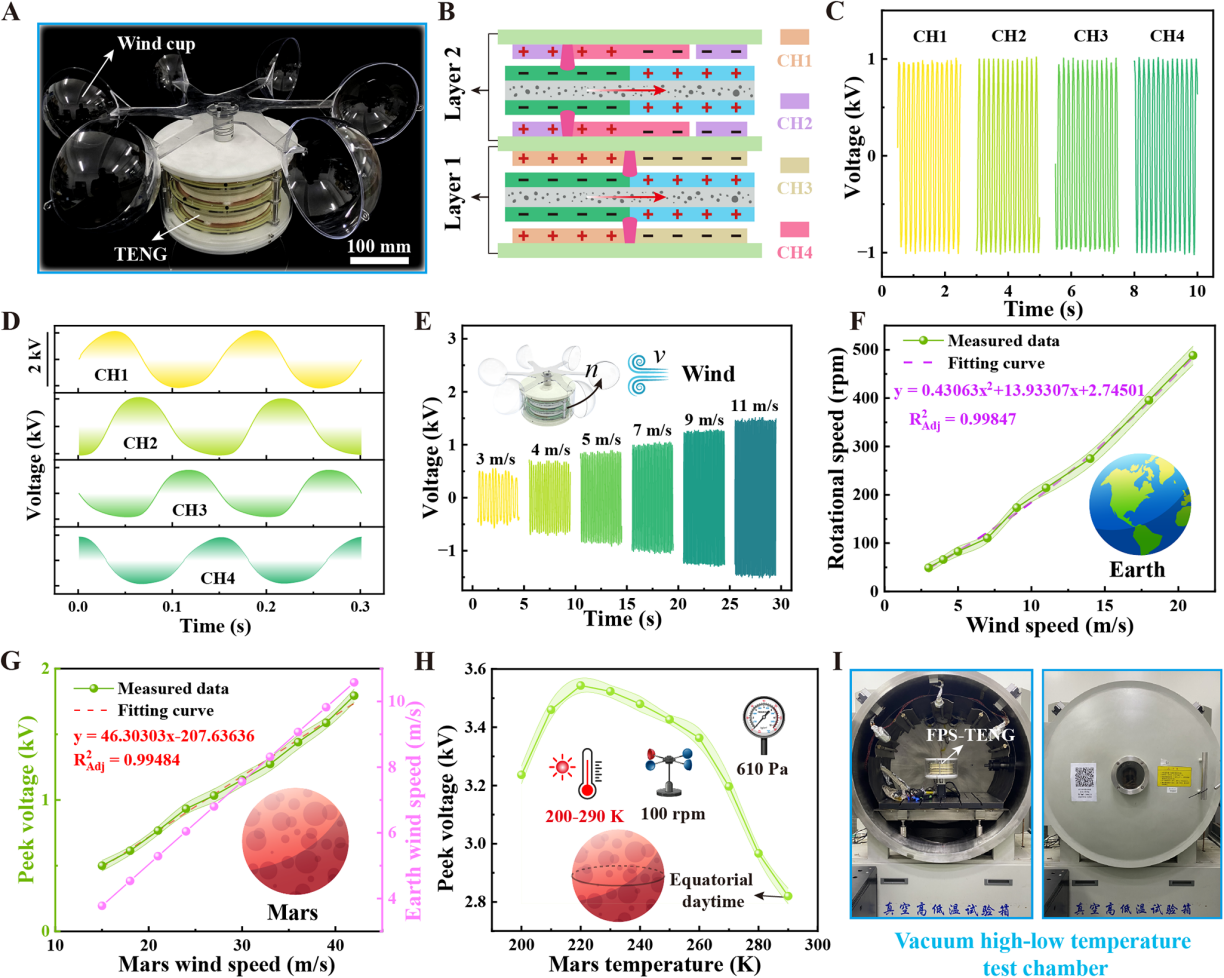
Output characteristics of the wind‐driven FPS‐TENG. A) A physical photograph of the wind‐driven FPS‐TENG. B) Schematic of a cross‐section of the FPS‐TENG at a specific instant. C,D) Schematic diagram of the voltage amplitude and phase output of the FPS‐TENG at a wind speed of 7 m s^−1^. E) The output voltage of the first phase in the FPS‐TENG across various wind speeds. F) Correlation between the FPS‐TENG's rotational speed and wind speed. G) The output voltage of the FPS‐TENG at different wind speeds within the Martian environment. H) Influence of temperature on the FPS‐TENG's output voltage under Martian atmospheric pressure conditions. I) Vacuum high‐low temperature experimental apparatus.

**Figure** **4**A displays a photograph of the FPS‐TENG, which incorporates an acrylic sphere as a substitute for a traditional wind cup, linked to the rotor through the rotating shaft. Figure 4B offers a schematic cross‐sectional view of the FPS‐TENG during operation, indicating that the upper stator electrodes CH2 and CH4 are positioned 22.5° from the lower stator electrodes CH1 and CH3. The two rotors are aligned precisely, enabling the FPS‐TENG to generate theoretical sinusoidal voltage signals with each phase differing by 90°. Figure 4C,D presents the actual output voltage signals, confirming that the amplitudes of the four phases are nearly identical and that the phase angles are ≈90° apart, aligning with the theoretical design effect (Movie S2, Supporting Information). Given the similar voltage amplitudes across phases, Phase 1 (CH1) output voltage is utilized for testing in this study.

Figure 4E illustrates the FPS‐TENG's output voltage at varying wind speeds on Earth, demonstrating that the voltage increases with higher wind speeds and that the device functions efficiently at a wind speed of 3 m s^−1^. Figure 4F explores the rotational speed of the FPS‐TENG under different wind conditions, illustrating a clear relationship between wind speed and rotor speed. To validate the FPS‐TENG's operational capacity within Martian wind speed ranges, its output voltage under various wind speeds was tested, as depicted in Figure 4G. Utilizing the conversion relationship between Earth and Martian wind speeds,^[^ ^37^ ^]^ a terrestrial wind speed of 3.75–10.57 m s^−1^ equates to Martian wind speeds of 15–42.28 m s^−1^. Given that the typical Martian wind speed ranges from 25.8‐41 m s^−1^,^[^ ^38^ ^]^ this indicates that the FPS‐TENG can effectively operate within Martian wind parameters.

The impact of Martian environmental factors (air pressure and temperature) on the output performance of the FPS‐TENG was investigated, as shown in Figure 4H. The tests were conducted in a vacuum high‐low temperature test chamber that mimics Martian air pressure and temperature conditions, as depicted in Figure 4I. Figure 4H reveals that the output voltage declines as the temperature rises. At the same temperature of 290 K and rotational speed of 100 rpm, the FPS‐TENG's output voltage under Martian air pressure (610 Pa^[^ ^39^ ^]^) reaches 2.81 kV, which is significantly higher than the 1.08 kV output under normal atmospheric pressure. Given that the temperature range on Mars spans 200–290 K,^[^ ^38^ ^]^ the FPS‐TENG is well‐suited for operation under the harsh Martian climate, potentially boosting output voltage.

### 2.4 Analysis of the Dust Removal Characteristics of the Four‐Phase Electrode

**Figure 5:**
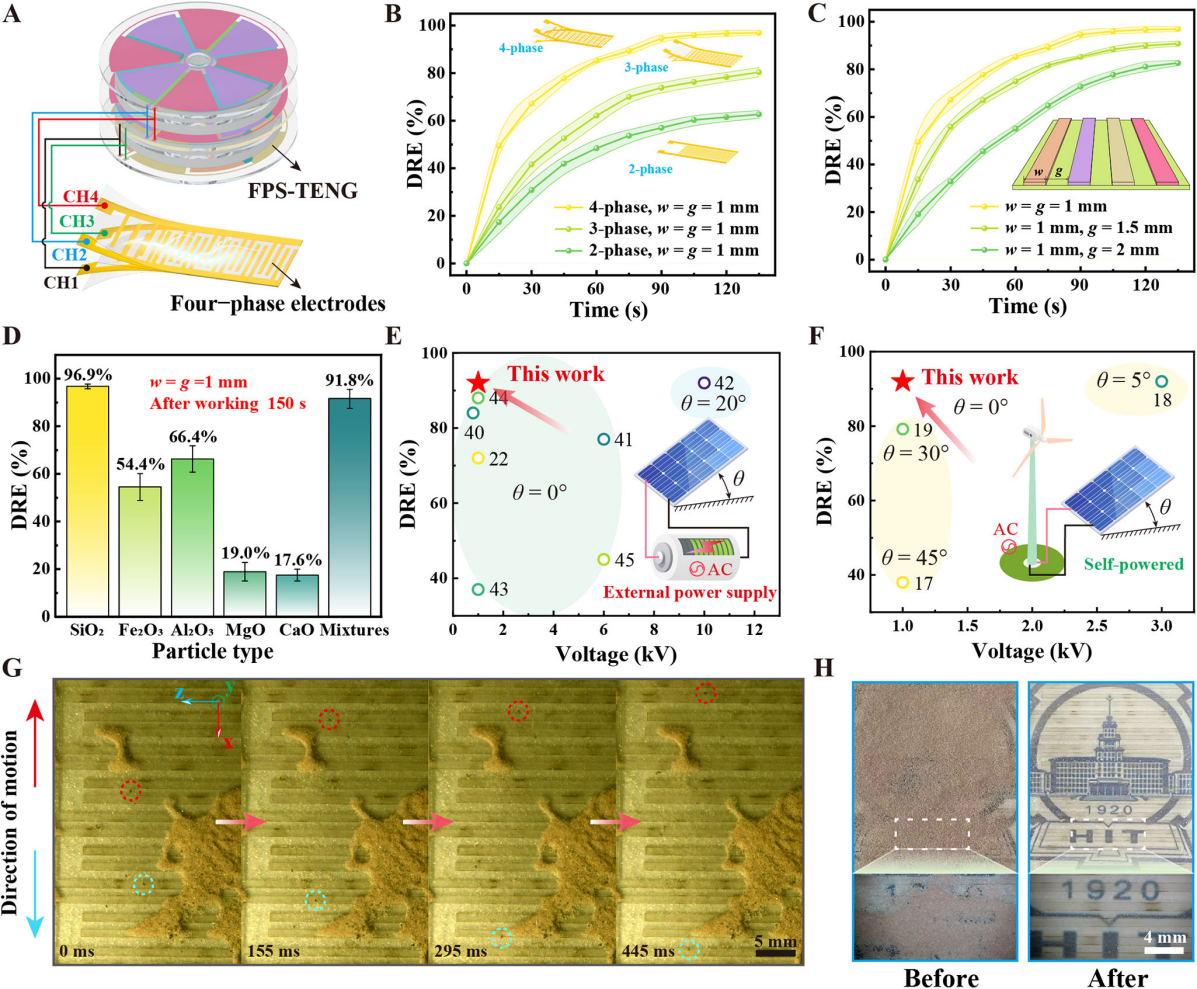
The impact of the four‐phase electrode structure parameters on the dust removal of FPS‐TENG. A) Schematic diagram of the connection between the four‐phase electrode and the FPS‐TENG. B) Effect of different phase numbers of the electrode on dust removal efficiency. C) Impact of different gap‐width ratios of the electrodes on dust removal efficiency. D) Impact of dust of different compositions on dust removal efficiency. E) Comparison of dust removal efficiency between the FPS‐TENG and dust removal devices powered by external power sources. F) Comparison of triboelectric dust removal efficiency. G) Trajectories of dust particles captured by a high‐speed camera. H) Comparison of the before and after effects of dust removal.

**Figure** **5**A illustrates the schematic of the dust removal device, comprising an FPS‐TENG and transparent four‐phase electrodes. The transmittance of the electrode structure at visible light wavelengths has been evaluated, revealing that the ITO interdigital electrodes coated with PET exhibit a transmittance exceeding 80% (Figure S9A, Supporting Information). The high light transmittance of the four‐phase electrodes minimally impacts solar panel performance, thereby preserving photovoltaic efficiency.

The width of the interdigital electrode's teeth denoted as *w*, and the gap between the teeth, denoted as *g*, are shown in Figure 1E. The impact of the number of electrode phases on dust removal efficiency (*DRE*), defined as *DRE* = Δ*m*/*m*_0_, where Δ*m*is the mass of dust removed and *m*_0_ is the initial dust mass, was also studied. The results are shown in Figure 5B. Specifically, Figures S10, S11 (Supporting Information) illustrate the wiring diagrams of two‐phase and three‐phase electrodes and their corresponding phase outputs of the TENGs. Their harmonic distributions are shown in Figure S12 (Supporting Information) and discussed in Note S2 (Supporting Information). Each experimental setup was conducted three times to ensure consistency. When *w* = *g*, the four‐phase electrode structure achieved the highest *DRE*, while the two‐phase and three‐phase electrodes exhibited lower efficiencies, leading to the selection of the four‐phase configuration for the dust removal electrode. To examine how the electrode's width‐to‐gap ratio (*η* = *w*/*g*) affects *DRE*, three sets of electrodes with varying ratios were tested, with the results presented in Figure 5C. The smaller the value of *η*, the greater the dust removal efficiency. When *η* = 1, the dust removal effect was superior to the other two groups, thus, the electrodes with *η* set to 1 were chosen. As shown in Figure S9B (Supporting Information), the dust removal results for three different *w* parameters when *η* = 1 reveal that the highest dust removal efficiency is achieved when *w* = *g* = 1 mm; therefore, the electrode structural parameters are set to *w* = *g* = 1 mm.

Figure S9C (Supporting Information) shows the impact of rotational speed on dust removal efficiency, indicating that higher rotational speeds enhance dust removal efficiency. In conjunction with Figure 3C, it is evident that greater output voltage correlates with higher dust removal efficiency. Additionally, we investigated the effect of relative humidity on dust removal efficiency. As shown in Figure S9D (Supporting Information), dust removal efficiency is higher in environments with lower relative humidity, while efficiency decreases as humidity increases. Martian dust, primarily composed of SiO_2_, Fe_2_O_3_, Al_2_O_3_, MgO, and CaO, is analyzed with their respective proportions shown in Figure S13A (Supporting Information) and their SEM images in Figure S13B (Supporting Information). Figure 5D examines the influence of different substances on dust removal efficiency, showing that the device achieves the highest efficiency for SiO_2_ particles and the lowest for CaO. The dust removal efficiency for Mars simulant dust (mixtures) reaches 91.8%. The efficiency of the FPS‐TENG presented in this study was compared with other dust removal devices that rely on external power supplies,^[^ ^22^, ^40^, ^41^, ^42^, ^43^, ^44^, ^45^ ^]^ showing that the FPS‐TENG can achieve higher dust removal efficiency at lower voltages, as shown in Figure 5E. Even when compared with other triboelectric (self‐powered) dust removal devices,^[^ ^17^, ^18^, ^19^ ^]^ as shown in Figure 5F, the FPS‐TENG removes dust efficiently at a low voltage without needing to tilt the solar panels. Therefore, the FPS‐TENG dust removal device designed in this study offers significant advantages.

High‐speed cameras were used to capture the motion of dust particles on the electrodes, as shown in Figure 5G. The dust particles move directionally along the positive and negative directions of the *x*‐axis of the electrodes under the influence of the traveling wave electric field, aligning with the analysis of the aforementioned Equations (2) and (3) (Movie S3, Supporting Information). Figure 5H demonstrates the dust removal effect on simulated Martian dust, where the Martian dust covers the background pattern, which is severely obscured and unrecognizable before dust removal. After dust removal, the background pattern becomes clearly visible. The dust accumulates on both sides of the electrodes (Figure S14, Movie S4, Supporting Information), illustrating the excellent dust removal effects of the device.

### 2.5 Wind‐Driven FPS‐TENG Dust Removal Demonstration for Mars Rover Solar Panels

**Figure 6:**
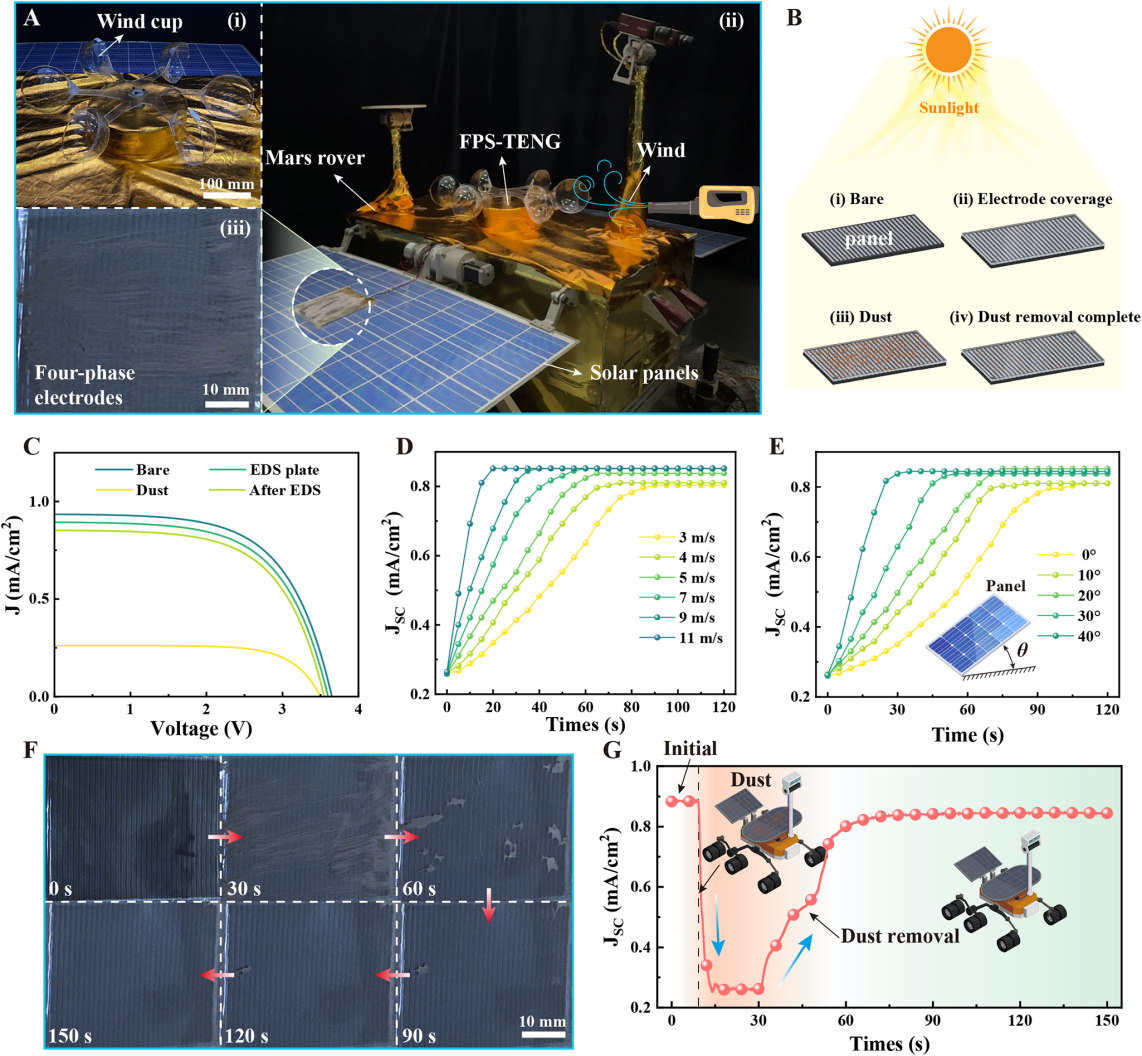
Demonstration of dust removal from Mars rover solar panels using the wind‐driven FPS‐TENG. A) Mars rover and its dust removal device. (i) Wind cup, (ii) Solar panel covered with dust, (iii) Actual installation photo of the dust removal device. B) Four states of solar panel illumination. C) Output curves of current density versus voltage for the solar panel under four states. D) Variation curve of short‐circuit current density *J*_SC_ of the solar panel over time at different wind speeds in the Earth's environment. E) Effect of different tilt angles of the solar panel on dust removal efficiency. F) Dust removal effect on the Mars rover solar panel over different time periods. G) Variation curve of short‐circuit current density *J*_SC_ of the solar panel during the dust removal process (wind speed: 7 m s^−1^).

To verify the dust removal capabilities of FPS‐TENG on Martian dust under real‐world conditions, as shown in **Figure** **6**A, a Martian rover dust removal system was constructed, utilizing larger‐sized electrodes (Figure S15, Supporting Information). FPS‐TENG was installed on the Mars rover (i‐ii), and wind conditions on Mars were mimicked using a blower. Electrodes for dust removal were positioned on the solar panels of the Mars rover, which were laden with Martian dust, as shown in Figure 6Aiii. Figure 6B illustrates the solar panels in four different conditions: i) the bare panel, ii) the panel covered with electrodes, iii) the panel covered with dust, and iv) the panel post‐dust removal. The output voltage and current density of the panel in these conditions were evaluated, as shown in Figure 6C. A higher short‐circuit current density *J*_SC_ correlates with fewer dust particles on the solar panel, enhancing the dust removal efficacy. The data from Figure 6C confirm that the bare panel demonstrates the highest *J*_SC_ and that dust coverage significantly diminishes it. After cleaning, the *J*_SC_ nearly restores to its original value (ii). Consequently, the electrodes placed for dust removal on the solar panels minimally impact the output performance of the solar panels and markedly improve dust removal.

The present study also explored how wind speed influences dust removal efficiency and effectiveness, with findings presented in Figure 6D. It was observed that higher wind speeds shortened the duration of dust removal, enhanced the dust removal efficiency, and improved the overall removal outcomes. In line with Figure 4G from the previous section, it is evident that effective dust removal is achievable within a Martian wind speed range of 15–42.28 m s^−1^. Adjusting the angle of the Mars rover's solar panels is typically necessary to optimize solar energy collection. Because the solar incidence angle and seasonal changes on Mars differ from those on Earth, it is crucial to adjust the panel angles to ensure they are optimally positioned relative to the sun, thus enhancing energy conversion efficiency. This necessitates the examination of how the tilt angle of the solar panels affects the dust removal effectiveness. As depicted in Figure 6E, the variation curves of the short‐circuit current density *J*_SC_ of the solar panels over time during dust removal at various inclination angles reveal that a steeper tilt angle results in higher dust removal efficiency and effectiveness.

Figure 6F illustrates the dust removal process at a wind speed of 7 m s^−1^ with the solar panel positioned horizontally. Within the initial 60 s, a significant amount of dust was removed from the panel, and by 150 s, the panel was entirely removed (Movie S5, Supporting Information). Figure 6G depicts the variation in the *J*_SC_ of the solar panel during the dust removal process. Initially, the panel recorded a current density of 0.88 mA cm^−2^, which decreased significantly as dust accumulated, substantially affecting solar power generation. 60 s into the dust removal, the *J*_SC_ quickly escalated, nearing 90% of its original value, indicating effective dust clearance. Subsequently, the *J*_SC_ continued to increase gradually, stabilizing at 0.84 mA cm^−^^2^. These results collectively demonstrate that FPS‐TENG is highly efficient and effective at removing dust from Mars rover solar panels within the studied Martian wind speed range.

## 3 Conclusion

This paper introduces a wind‐driven ternary four‐phase rotary triboelectric nanogenerator, designated as FPS‐TENG, for self‐powered dust removal on solar panels during Mars exploration missions. The FPS‐TENG employs a double‐layer stacked structure that significantly enhances the output voltage via a soft‐soft contact mechanism. The FPS‐TENG achieved a dust removal efficiency of up to 91.8% under simulated Martian wind conditions and demonstrated stability and durability in tests simulating the extreme Martian environment. The adaptability of the FPS‐TENG to various wind speeds, temperatures, and panel tilt angles was also verified. Tests indicated that this technology maintains high efficiency in dust removal without dependence on the tilt angle of the solar panels, even at lower voltages, underscoring its potential for space exploration applications. Due to equipment limitations, we were unable to conduct full‐scale experimental tests. Future work will include broader size testing and validation in practical application scenarios. With continued development and refinement, the FPS‐TENG technology holds significant promise for application in Mars exploration and a wider array of deep space missions.

## 4 Experimental Section

### Materials

Nylon film was sourced from the Wan Da Filtration Equipment Business Department in Haining City. PTFE film was obtained from Chenguang Plastic Industry Co., Ltd. in Taizhou City. Polyester fur was acquired from Jinjin Textile Co., Ltd. in Pujiang County. ITO conductive film was procured from Xingyuan Technology Co., Ltd. in Huizhou City.

### Fabrication of the FPS‐TENG

The stator configuration includes Cu sector‐shaped electrode plates mounted on an acrylic base plate. Using Printed Circuit Board technology, the electrode plates were crafted with two sets of sector‐shaped electrodes on the PCB, each set separated by a 4 mm gap. An acrylic plate, 4 mm in thickness, was laser‐cut into a disk measuring 190 mm in outer diameter and 17 mm in inner diameter. These electrodes were then affixed to the base plate, with polyester fur positioned in the gaps to complete the stator assembly.

The rotor consists of a central acrylic plate, sponges on either side, and was covered with PTFE and nylon films shaped to fit. Initially, sponges were attached to each side of the plate. The PTFE and nylon films, cut into fan shapes, were then adhered to the sponges.

The assembly process involved aligning the stator and rotor into a single‐layer FPS‐TENG with a 2 mm separation. For the double‐layer version, the components were adjusted to have a phase angle difference of 22.5°. Finally, the rotor was connected to a wind cup made from six acrylic balls using a rotating shaft, completing the assembly.

### Fabrication of the Four‐Phase Electrode

A picosecond laser was used to precision‐cut ITO film, 0.125 mm thick, into interdigitated electrodes. These electrodes were then affixed to a 0.05 mm thick PET film on both sides. A protective PET film was subsequently placed over the electrodes to finalize the four‐phase electrode.

### Measurement

The open‐circuit voltage (*V*_OC_) of the FPS‐TENG was measured with a digital oscilloscope (Tektronix 3 SERIES). The short‐circuit current (*I*_SC_) and transferred charge (*Q*_SC_) were relayed to a computer through a programmable electrostatic voltmeter (6514, Keithley, USA) and a data acquisition system (PCI‐6289, National Instruments, USA), with results displayed and logged via LabVIEW software. The structural and surface characteristics of the PTFE, nylon films, and polyester fur, along with dust particle sizes, were examined using a scanning electron microscope (TESCAN CLARA). An industrial‐grade high‐speed camera (PHANTOM V12.1) recorded the dust particles' motion trajectories.

## Conflict of Interest

The authors declare no conflict of interest.

## Author Contributions

F.Y., Z.X., J.X., and X.H. conceived the project. Z.X. and J.X. managed the research and provided crucial suggestions for the main concept of the project. F.Y., Z.W., and J.X. prepared the triboelectric nanogenerator dust removal device. Z.W., B.X., Y.L., and X.H. analyzed the data. F.Y., Z.W., and J.X. designed the figures and wrote the manuscript. All authors contributed to editing the manuscript. Z.X., J.X., and X.H. supervised the research.

## Supporting information



Supporting Information

Supplemental Movie 1

Supplemental Movie 2

Supplemental Movie 3

Supplemental Movie 4

Supplemental Movie 5

## Data Availability

The data that support the findings of this study are available from the corresponding author upon reasonable request.
