# Supplementary material for: A Soft‐Soft Contact Triboelectric Nanogenerator with a Ternary Four‐Phase Structure for Self‐Powered High‐Efficiency Dust Removal on Mars
Source: Adv Sci (Weinh). 2025 Apr 17;12(27):2502956. doi: 10.1002/advs.202502956 (PMC12279170; doi:10.1002/advs.202502956)
Supplement: Supplementary file 1 — Supporting Information [file ADVS-12-2502956-s006.docx]

Supporting Information

**A Soft-Soft Contact Triboelectric Nanogenerator with a Ternary Four-Phase Structure, for Self-Powered High-Efficiency Dust Removal on Mars**

**Fei Yang^a^, Zheping Wang^a^, Boyi Xu^b^, Yifan Lu^a^, Xuyan Hou^a,^*, Jinsui Xu^a,^*, Zhijie Xie^c,^***

^a^National Key Laboratory of Aerospace Mechanism, School of Mechatronics Engineering, Harbin Institute of Technology, Harbin, 150001, China

^b^Wu Xianming School of Intelligent Engineering, South China University of Technology, Guangzhou, 510641, China

^c^College of mechanical and electrical engineering, Northeast Forestry University, Harbin, 150042, China

*Corresponding authors’ e-mail address: houxuyan@hit.edu.cn (X.H.); [xjs127@126.com](mailto:xjs127@126.com) (J.X.);

[xiezhijie111@sina.com](mailto:xiezhijie111@sina.com) (Z.X.)

**Supporting Notes**

**Note S1: Equivalent diagram of charge transfer.**

As shown in Figure S6, using the principle of potential superposition to analyze the working mechanism of an independent layer TENG, for a TENG containing only a PTFE film, we first assume that only a small interface d*θ* on the surface of the friction layer sector contains triboelectric charges, with a charge density of -*σ*_1_. The total charge induced by this small interface on copper electrodes A and B can be approximated as (*R*_2_^2^-*R*_1_^2^)*σ*_1_d*θ*/2. The capacitances between the small interface and copper electrodes A and B are denoted as *C_A_*(*α*) and *C_B_*(*α*), respectively. Under short-circuit conditions, the charges on copper electrodes A and B (dQ_A1_ and dQ_B1_) can be expressed by the following equations:

$$dQ_{A1}=\frac{C_{A}\left(\alpha\right)}{C_{A}\left(\alpha\right)+C_{B}\left(\alpha\right)}\cdot\frac{(R_{\mathbf{2}}^{\mathbf{2}}-R_{\mathbf{1}}^{\mathbf{2}})\sigma_{1}d\theta}{2}=\frac{(R_{\mathbf{2}}^{\mathbf{2}}-R_{\mathbf{1}}^{\mathbf{2}})\sigma_{1}d\theta}{1+\frac{C_{B}\left(\alpha\right)}{C_{A}\left(\alpha\right)}}$$

$$dQ_{B1}=\frac{C_{B}\left(\alpha\right)}{C_{A}\left(\alpha\right)+C_{B}\left(\alpha\right)}\cdot\frac{(R_{\mathbf{2}}^{\mathbf{2}}-R_{\mathbf{1}}^{\mathbf{2}})\sigma_{1}d\theta}{2}=\frac{(R_{\mathbf{2}}^{\mathbf{2}}-R_{\mathbf{1}}^{\mathbf{2}})\sigma_{1}d\theta}{1+\frac{C_{A}\left(\alpha\right)}{C_{B}\left(\alpha\right)}}$$

Based on the principle of electrostatic field superposition, considering that the dielectric surface consists of four sectors, the total charge is the sum of the contributions from each electrostatic region. The total charges on copper electrodes A and B can be expressed as:

$$Q_{A1}=2(R_{\mathbf{2}}^{\mathbf{2}}-R_{\mathbf{1}}^{\mathbf{2}})\sigma_{1}\int_{0}^{\frac{\pi}{4}} \frac{d\theta}{1+\frac{C_{B}\left(\alpha\right)}{C_{A}\left(\alpha\right)}}$$

$$Q_{B1}=2(R_{\mathbf{2}}^{\mathbf{2}}-R_{\mathbf{1}}^{\mathbf{2}})\sigma_{1}\int_{0}^{\frac{\pi}{4}} \frac{d\theta}{1+\frac{C_{A}\left(\alpha\right)}{C_{B}\left(\alpha\right)}}$$

For a TENG containing only a Nylon film, the sector positions are complementary to those of the PTFE film. The total charge induced by a small interface can be approximated as -(*R*_2_^2^-*R*_1_^2^)*σ*_2_d*θ*/2. The capacitances between the small interface and copper electrodes A and B are denoted as *C_A_*(***π*/4-***α*) and *C_B_*(***π*/4-***α*), respectively. It can be determined that *C_A_*(***π*/4-***α*) = *C_B_*(*α*) and *C_B_*(***π*/4-***α*) = *C_A_*(*α*). Thus, the total charges induced by the Nylon film on copper electrodes A and B can be expressed as:

$$Q_{A2}=-2(R_{\mathbf{2}}^{\mathbf{2}}-R_{\mathbf{1}}^{\mathbf{2}})\sigma_{2}\int_{0}^{\frac{\pi}{4}} \frac{d\theta}{1+\frac{C_{A}\left(\alpha\right)}{C_{B}\left(\alpha\right)}}$$

$$Q_{B2}=-2(R_{\mathbf{2}}^{\mathbf{2}}-R_{\mathbf{1}}^{\mathbf{2}})\sigma_{2}\int_{0}^{\frac{\pi}{4}} \frac{d\theta}{1+\frac{C_{B}\left(\alpha\right)}{C_{A}\left(\alpha\right)}}$$

Therefore, when the rotor's movement angle equals the sector angle of 45°, the transferred charge *Q*_SC_ of the TENG during this period can be expressed as:

$$\begin{matrix} Q_{SC}=\left.\left(Q_{A1}+Q_{A2}\right)\right|_{\alpha=\frac{\pi}{4}}-\left.\left(Q_{A1}+Q_{A2}\right)\right|_{\alpha=0} \\ \text{ }=2(R_{\mathbf{2}}^{\mathbf{2}}-R_{\mathbf{1}}^{\mathbf{2}})\left(\sigma_{1}+\sigma_{2}\right)\left(\int_{0}^{\frac{\pi}{4}} \frac{d\theta}{1+\frac{C_{A}\left(0\right)}{C_{B}\left(0\right)}}-\int_{0}^{\frac{\pi}{4}} \frac{d\theta}{1+\frac{C_{B}\left(0\right)}{C_{A}\left(0\right)}}\right) \end{matrix}$$

**Note S2. Harmonic voltage distribution on EDS.**

As shown in Figure S12A, for the two-phase electrodes (*n* = 2):

$$\begin{matrix} V_{2}\left(x,y,t\right)\approx V_{0}A_{21}e^{-\frac{2\pi}{\lambda_{2}}y}\cos\left(\frac{2\pi}{\lambda_{2}}x\right)\cos\left(\omega t\right) \\ \text{ }=V_{0}\left[\frac{A_{21}}{2}e^{-\frac{2\pi}{\lambda_{2}}y}\cos\left(\frac{2\pi}{\lambda_{2}}x-\omega t\right)+\frac{A_{21}}{2}e^{-\frac{2\pi}{\lambda_{2}}y}\cos\left(\frac{2\pi}{\lambda_{2}}x+\omega t\right)\right] \end{matrix}$$

$$V_{2for}\left(y\right)=V_{2back}\left(y\right)=\frac{A_{21}}{2}e^{-\frac{2\pi}{\lambda_{2}}y}$$

Where *V*_2_*_for_* (*y*) represents the amplitude of the forward wave and *V*_2_*_back_* (*y*) represents the amplitude of the backward wave. It can be seen that the forward and backward waves possess equal amplitudes and propagate in opposite directions, thereby resulting in the formation of a standing wave in the two-phase electrodes.

For *n* *≥* 3 phases, the electrode potential distribution can be decomposed using a Fourier series. In this decomposition, the fundamental component (also referred to as the first harmonic) and the (*n*-1)th harmonic exhibit notably higher amplitudes, while the amplitudes of the remaining harmonics are typically very small^[1,2]^. The potential distribution is expressed in Equation (10).

$$V_{n}(x,y,t)\approx V_{0}\cdot\left(\begin{matrix} A_{1}e^{-\frac{2\pi}{\lambda_{n}}y}\cos(\frac{2\pi}{\lambda_{n}}x-\omega t)+ \\ A_{n-1}e^{-\frac{2\left(n-1\right)\pi}{\lambda_{n}}y}\cos(\frac{2\left(n-1\right)\pi}{\lambda_{n}}x+\omega t) \end{matrix}\right),n\geq3$$

In which *A*_1_ and *A_n_*_-1_ are the Fourier coefficients for the forward and backward waves, respectively. Moreover, the exponential term in Equation (10) indicates that the potential decays exponentially along the y-direction.

As shown in Figure S12B, for the three-phase electrodes (*n* = 3), Equation (10) yields:

$$\begin{matrix} V_{3}\left(x,y,t\right)\approx W_{3for}+W_{3back} \\ \text{ }=V_{0}\left[A_{31}e^{-\frac{2\pi}{\lambda_{3}}y}\cos\left(\frac{2\pi}{\lambda_{3}}x-\omega t\right)+A_{32}e^{-\frac{4\pi}{\lambda_{3}}y}\cos\left(\frac{4\pi}{\lambda_{3}}x+\omega t\right)\right] \end{matrix}$$

$$\left\{\begin{matrix} V_{3for}\left(y\right)=V_{0}A_{31}e^{-\frac{2\pi}{\lambda_{3}}y} \\ V_{3back}\left(y\right)=V_{0}A_{32}e^{-\frac{4\pi}{\lambda_{3}}y} \end{matrix}\right.$$

Where *V*_3_*_for_* (*y*) denotes the amplitude of the forward wave and *V*_3_*_back_* (*y*) denotes the amplitude of the backward wave.

As shown in Figure S12C, for the four-phase electrodes (*n* = 4), Equation (10) yields:

$$\begin{matrix} V_{4}\left(x,y,t\right)\approx W_{4for}+W_{4back} \\ \text{ }=V_{0}\left[A_{41}e^{-\frac{2\pi}{\lambda_{4}}y}\cos\left(\frac{2\pi}{\lambda_{4}}x-\omega t\right)+A_{43}e^{-\frac{6\pi}{\lambda_{4}}y}\cos\left(\frac{6\pi}{\lambda_{4}}x+\omega t\right)\right] \end{matrix}$$

$$\left\{\begin{matrix} V_{4for}\left(y\right)=V_{0}A_{41}e^{-\frac{2\pi}{\lambda_{4}}y} \\ V_{4back}\left(y\right)=V_{0}A_{43}e^{-\frac{4\pi}{\lambda_{4}}y} \end{matrix}\right.$$

Where *V*_4_*_for_* (*y*) denotes the amplitude of the forward wave, and *V*_4_*_back_* (*y*) denotes the amplitude of the backward wave.

**Supporting Figures**


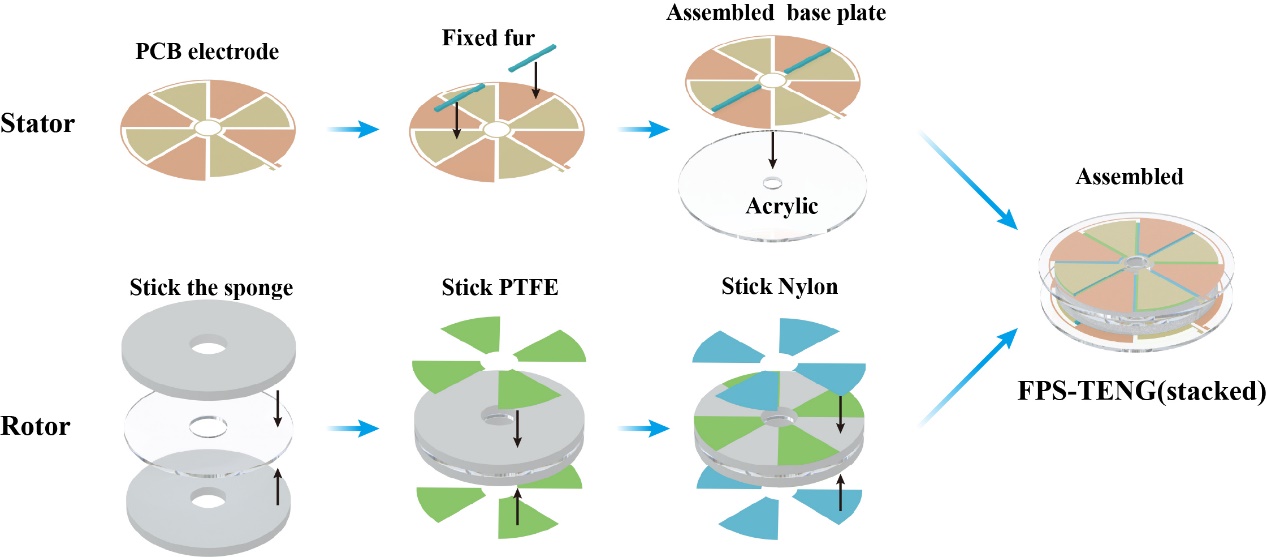


**Figure S1. Fabrication process of the FPS-TENG stator and rotor.**


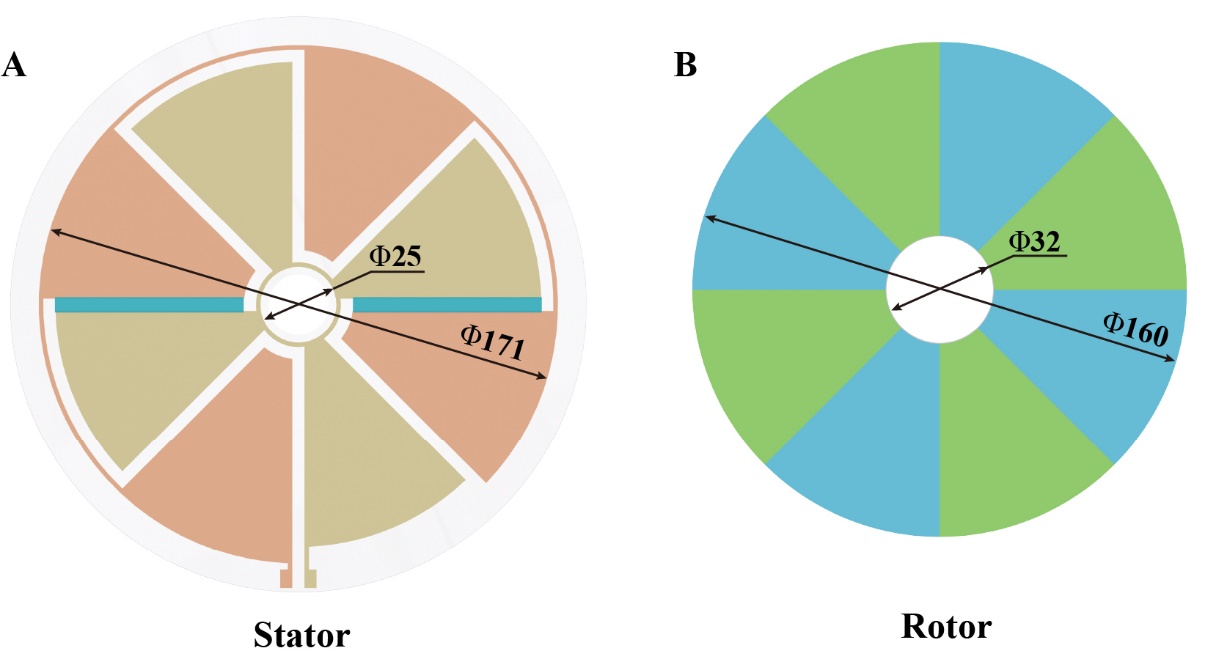


**Figure S2. Dimensions of the stator and rotor (**in millimeters)**.**


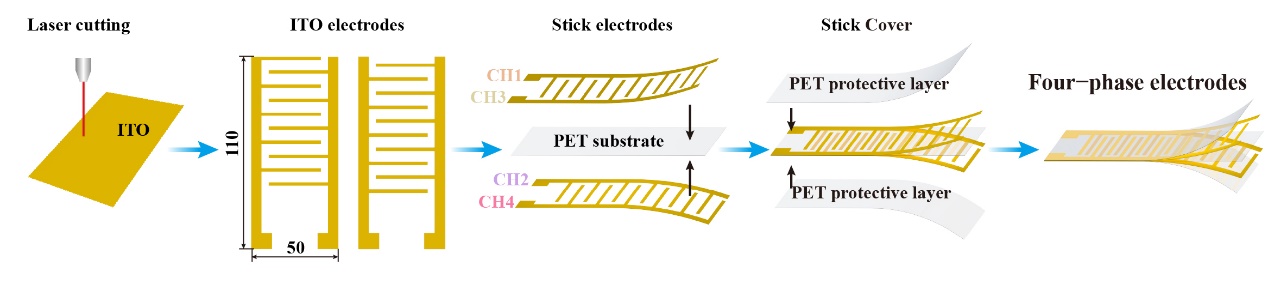


**Figure S3. Fabrication process of the four-phase electrode (**in millimeters)**.**


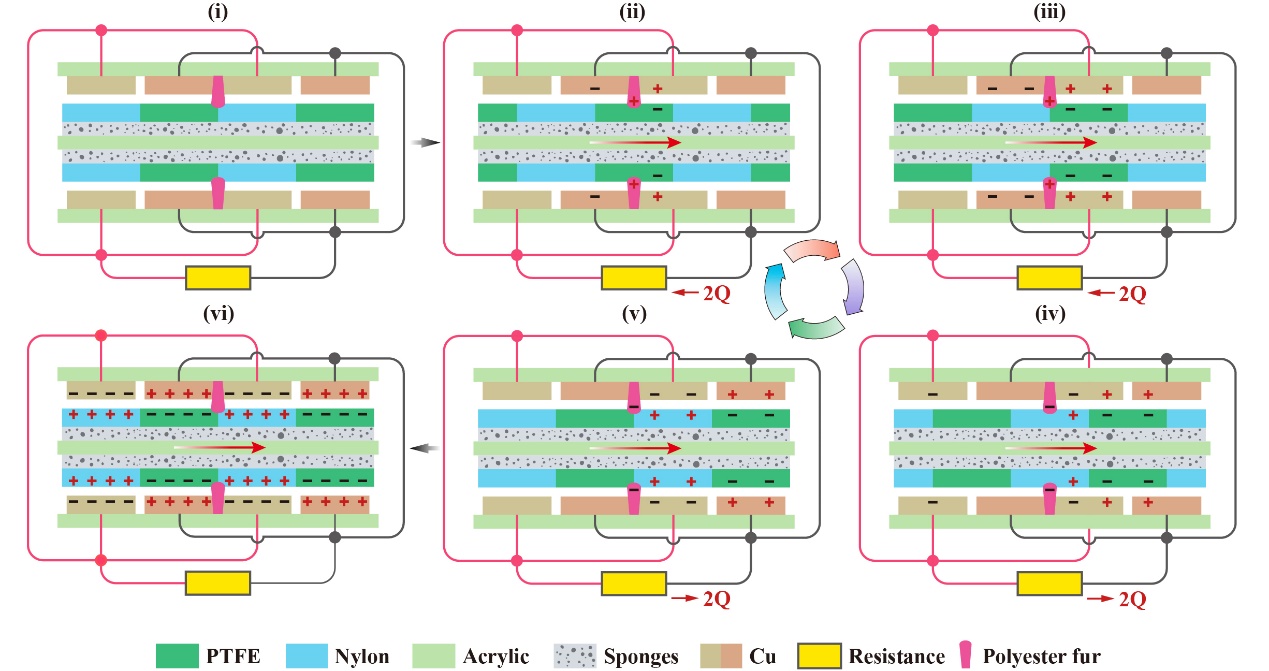


**Figure S4. Working principle of the FPS-TENG prior to charge saturation in the dielectric layer.** (i) Initial state, (ii-v) Progression to charge saturation, (vi) Schematic upon achieving charge saturation. Figure S4 illustrates the working principle of the generator unit before the dielectric layers reach saturation. In the initial state, the polyester brush on the rotor does not move relative to the upper and lower stators, and no charges are generated, as shown in Figure S4(i). Once the rotor begins to rotate, as shown in Figure S4(ii), the brushes on the upper and lower stator layers rub against the PTFE film. Since the brushes have higher electronegativity than the PTFE film, the brushes lose electrons and become positively charged, while the PTFE film gains electrons and becomes negatively charged. As the rotor continues to rotate, due to electrostatic induction, the copper electrodes corresponding to the PTFE film are induced with an equal amount of opposite charges, as shown in Figure S4(iii). When the brushes rub against the Nylon film during rotation, the brushes have higher electronegativity than the Nylon film, causing the Nylon film to lose electrons, which are transferred to the brushes, as shown in Figure S4(iv). As the rotor continues to rotate, due to electrostatic induction, the copper electrodes corresponding to the Nylon film is induced with an equal amount of opposite charges, as shown in Figure S4(v). After a period of cycling, the charges on the PTFE and Nylon films become saturated, as illustrated in Figure S4(vi). Before the charges reach saturation, the brushes act as an intermediate medium for charge transfer.


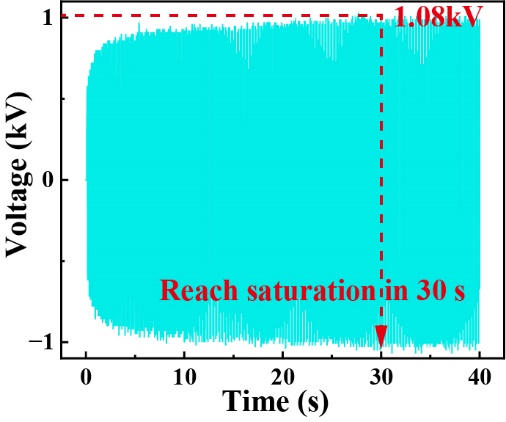


**Figure S5. Stabilization process of FPS-TENG voltage output.**

**
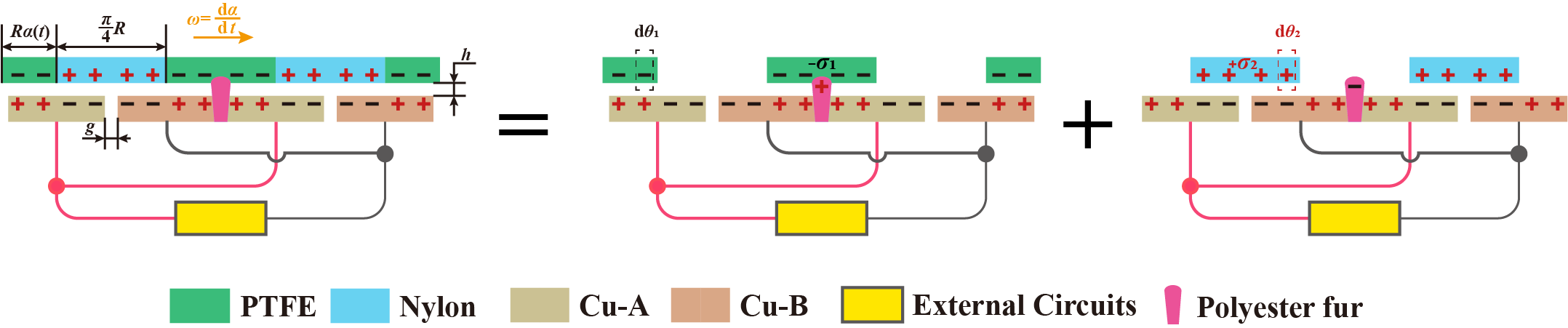
**

**Figure S6. Equivalent diagram of charge transfer.** For the FPS-TENG, during stable operation, it can be equivalently represented as two independent and complementary TENG layers working simultaneously. In the diagram, the distance between copper electrodes A and B is *g*, the gap between the copper electrodes and the friction layer material is *h*, the inner radius of the friction layer sector is *R*_1_, and the outer radius is *R*_2_. The charge density on the PTFE film is -*σ*_1_, and the charge density on the Nylon film is +*σ*_2_.


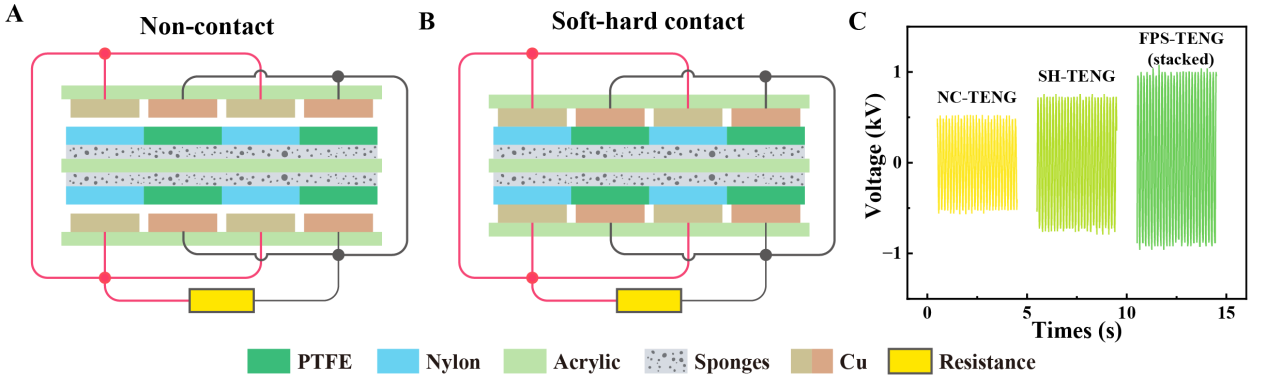


**Figure S7.** (**A**) Non-contact. (**B**) Soft-hard contact. (**C**) Comparison of outputs between non-contact, soft-hard contact, and FPS-TENG (stacked). Figure S7A shows the non-contact structure without polyester fur, and Figure S7B shows the contact structure without polyester fur. The results indicate that the output voltage of the FPS-TENG (stacked) with polyester fur is consistently higher than that of the structures without polyester fur.

**
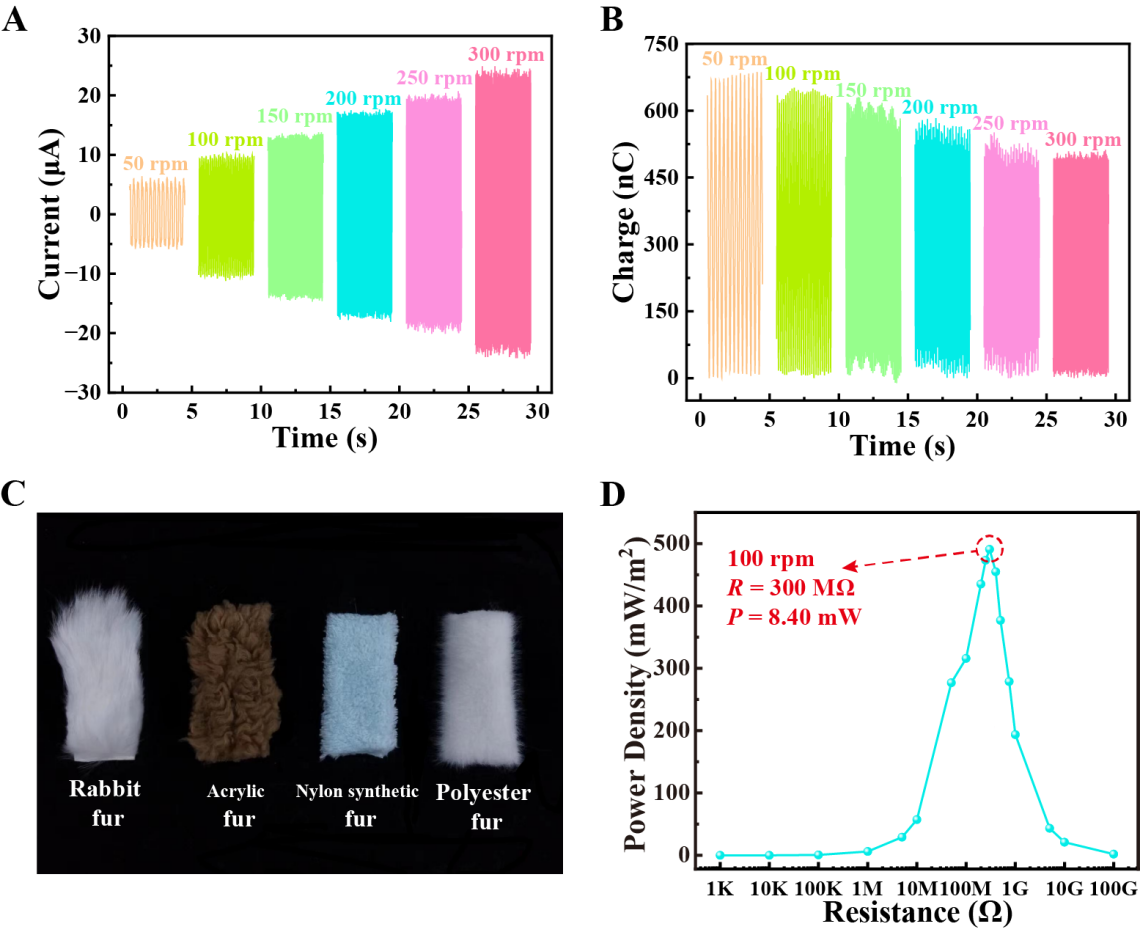
**

**Figure S8.** (**A**) Current and transferred charge of the FPS-TENG at different rotational speeds. (**B**) Transferred charge of the FPS-TENG at various rotational speeds. (**C**) Fur samples from different materials. (**D**) The power output of the FPS-TENG under different external loads.

**
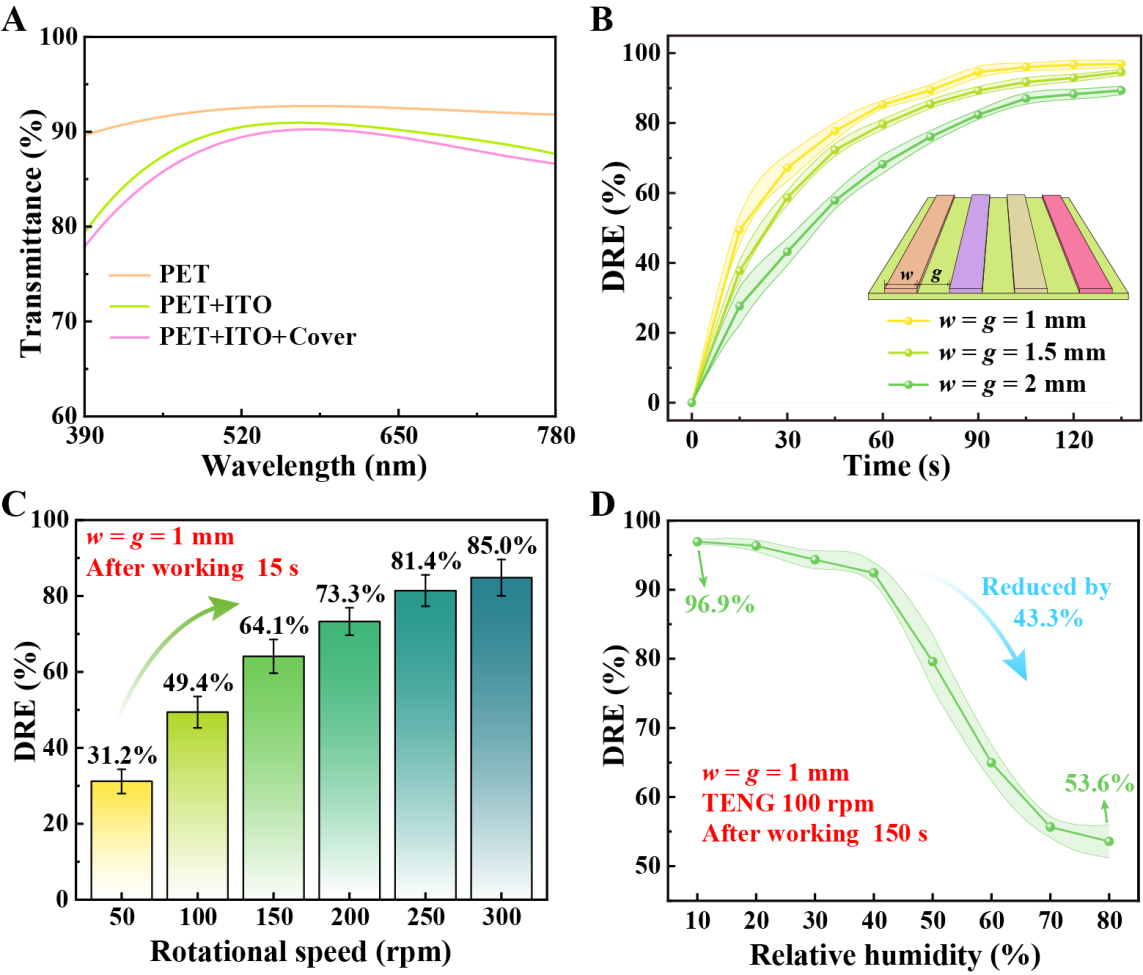
**

**Figure S9.** (**A**) Light transmittance of electrodes with different structures under visible light. (**B**) Effect of electrode configurations on dust removal at constant width-to-gap ratios. (**C**) Dust removal efficiency of the FPS-TENG at various rotational speeds. (D) Dust removal efficiency of the FPS-TENG at various relative humidity.

**
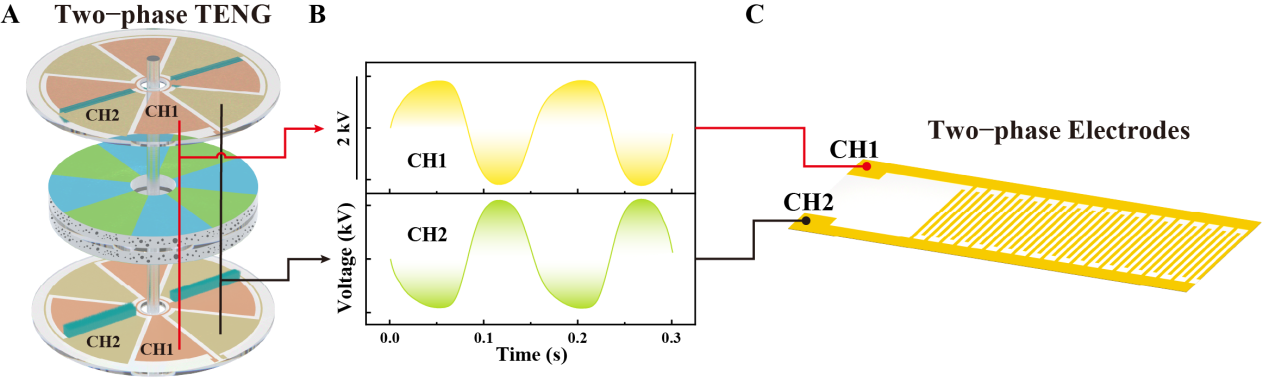
**

**Figure S10.** **Schematic diagram of the two-phase TENG structure and its electrode wiring.** (A) Two-phase stacked TENG structure diagram. (B) Schematic diagram of the output voltage amplitude and phase of the two-phase TENG under a wind speed of 7 m/s. (C) Schematic diagram of the wiring of the two-phase electrode structure.

**
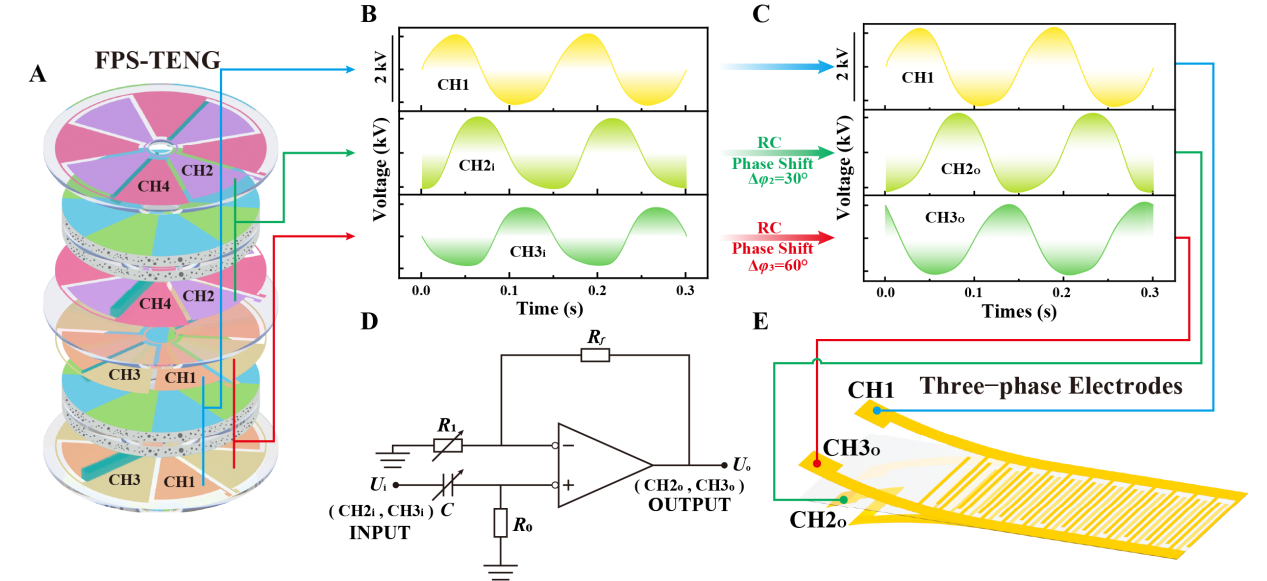
Figure S11.** **Schematic diagram of the wiring of the three-phase electrodes.** (A) FPS-TENG stacked structure diagram. (B) Schematic diagram of the output voltage amplitude and phase of phases 1-3 of the FPS-TENG under a wind speed of 7 m/s. (C) Schematic diagram of the output voltage amplitude and phase of phases 1-3 of the TENG after phase modification. (D) Schematic diagram of the phase shift circuit. (E) Schematic diagram of the wiring of the three-phase electrodes.

For the three-phase electrodes, we use the first three-phase outputs of the FPS-TENG, as shown in Figures S11(A-B). Since the original TENG outputs three-phase voltages with phase angles of 90º, the phase angles need to be adjusted to 120º (Figure S11C). Therefore, a phase-shifting circuit (Figure S11D) is employed to change the phase of CH2 from 90º to 120º and the phase of CH3 from 180º to 240º, after which they are connected respectively to the three pins of the three-phase electrodes. The principle of the RC phase shift circuit is as follows:

$$U_{O}=\left(1+\frac{R_{f}}{R_{1}}\right)\cdot\frac{R_{0}}{R_{0}+\frac{1}{j\omega C}}\cdot U_{i}$$

From Equation (16), we obtain:

In the above equation, *U_i_* is the input voltage of the phase-shifting circuit, *U_o_* is the output voltage, *A_u_* is the amplitude ratio between them, and Δ*φ* is the phase difference. *R*_0_ is the fixed resistor, *R*_1_ is the ratio resistor, *R_f_* is the feedback resistor, and *C* is the adjustable capacitor. By adjusting *R*_1_ and *C*, the phase can be tuned.

Initially, the capacitor *C* is adjusted so that the phase angles of CH2 and CH3 are altered by Δ*φ*_2_=30° and Δ*φ*_3_=60°, respectively. Subsequently, the resistance *R*_1_ is tuned to compensate for the amplitude attenuation caused by the phase adjustment, thereby ensuring that the output amplitude remains consistent with the original signal. As a result, the final inputs CH2_o_ and CH3_o_ for the three-phase electrodes are obtained.

**
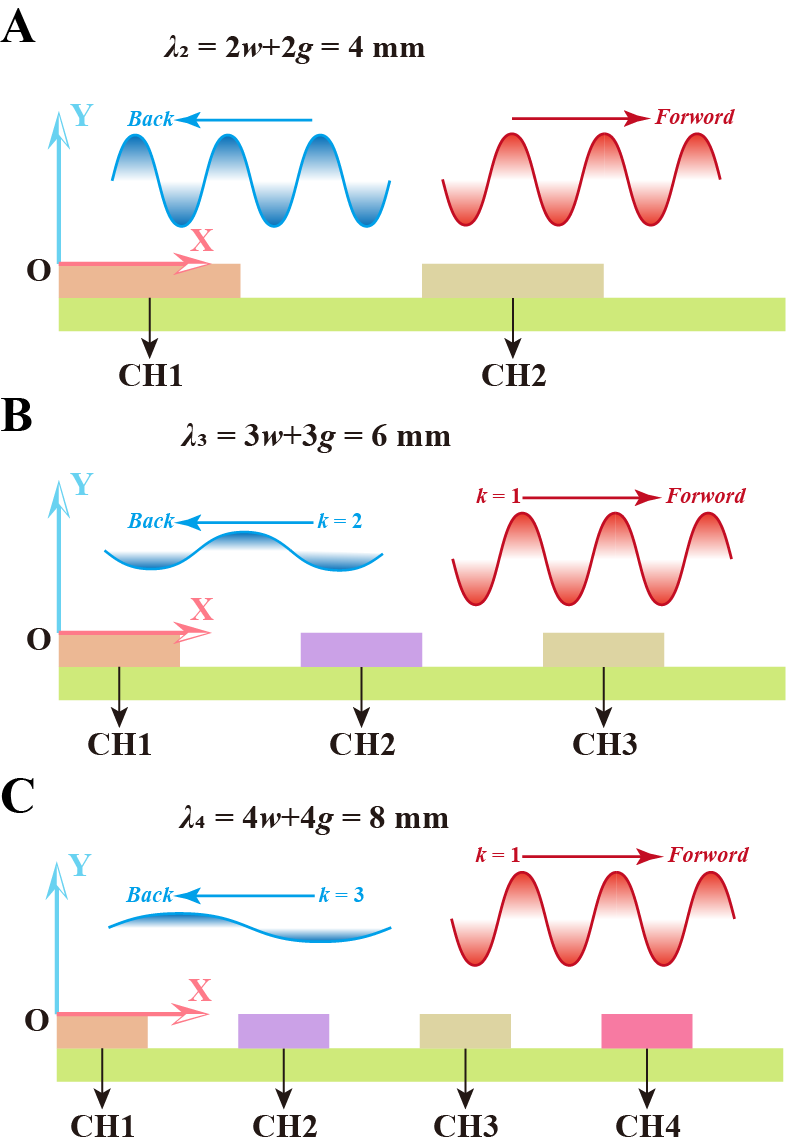
**

**Figure S12.** **(A-C) Schematic diagrams of the harmonic distribution on the two-, three-, and four-phase electrodes.**


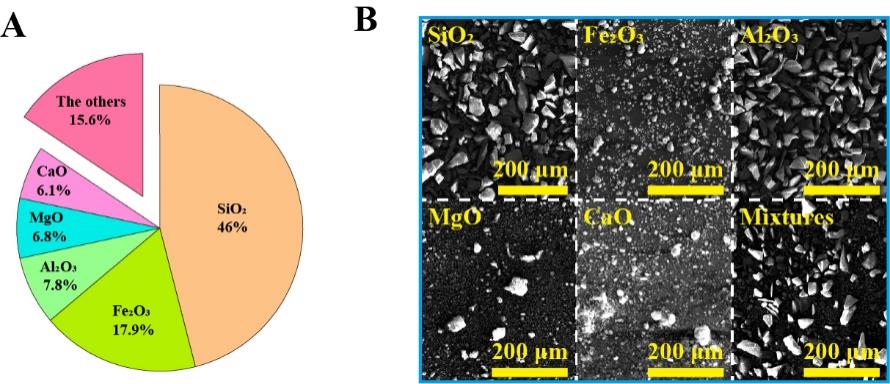


**Figure S13.** (**A**) Proportional distribution of different substances in Martian dust. (**B**) SEM images of various types of dust particles.


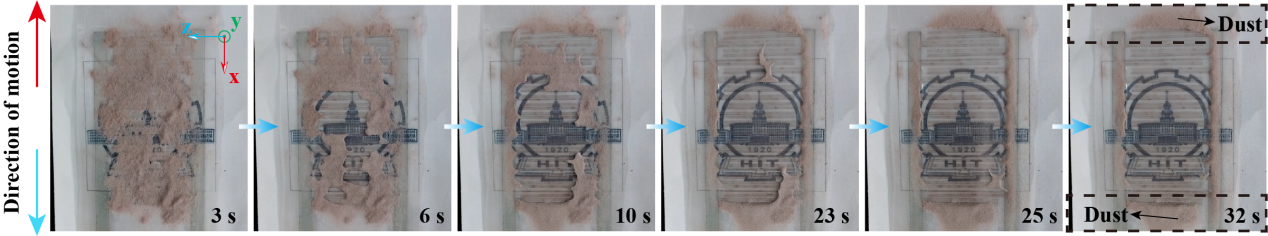


**Figure S14. The directional movement of dust in the traveling wave electric field.** The dust moves directionally along both the positive and negative x-axis directions, eventually accumulating on both sides of the electrodes.


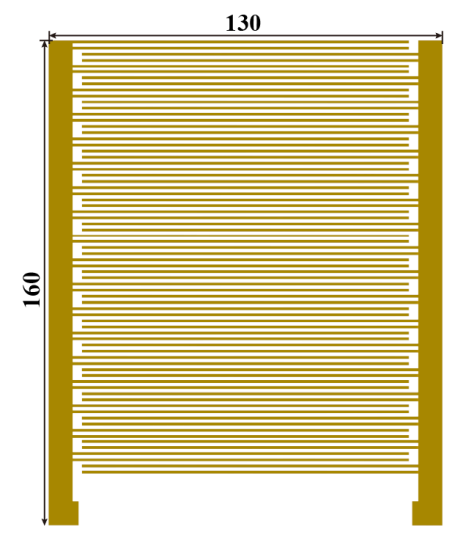


**Figure S15.** **Schematic diagram of large-sized electrodes (**in millimeters).

**Reference:**

[1] A. Zouaghi, N. Zouzou, J ELECTROSTAT 2019, 98, 25.

[2] S. Masuda, M. Washizu, M. Iwadare, IEEE T IND APPL 2008, 474.
